# A TMVP1-modified near-infrared nanoprobe: molecular imaging for tumor metastasis in sentinel lymph node and targeted enhanced photothermal therapy

**DOI:** 10.1186/s12951-023-01883-6

**Published:** 2023-04-17

**Authors:** Xueqian Wang, Geyang Dai, Guiying Jiang, Danya Zhang, Ling Wang, Wen Zhang, Huang Chen, Teng Cheng, Ying Zhou, Xiao Wei, Fei Li, Ding Ma, Songwei Tan, Rui Wei, Ling Xi

**Affiliations:** 1 Department of Gynecological Oncology, Tongji Hospital, Tongji Medical College, Huazhong University of Science and Technology, Wuhan, 430000 China; 2 National Clinical Research Center for Obstetrics and Gynecology, Cancer Biology Research Center (Key Laboratory of the Ministry of Education), Tongji Hospital, Tongji Medical College, Huazhong University of Science and Technology, Wuhan, 430000 China; 3 Department of Gynecology, West China Second University Hospital, Chengdu, 610000 China; 4 Tongji School of Pharmacy, Tongji Medical College, Huazhong University of Science and Technology, Wuhan, 430000 China; 5 Hubei University of Medicine, Shiyan, 442000 China; 6 School of Medicine, Jianghan University, Wuhan, 430000 China; 7 Department of Thyroid and Breast Surgery, Tongji Hospital, Tongji Medical College, Huazhong University of Science and Technology, Wuhan, 430030 China

**Keywords:** Molecular imaging, TMVP1, Sentinel lymph node, Photothermal therapy

## Abstract

**Background:**

TMVP1 is a novel tumor targeting polypeptide screened by our laboratory with a core sequence of five amino acids LARGR. It specially binds to vascular endothelial growth factor receptor-3 (VEGFR-3), which is mainly expressed on neo-lymphatic vessels in sentinel lymph node (SLN) with tumor metastasis in adults. Here, we prepared a targeted nanoprobe using TMVP1-modified nanomaterials for tumor metastasis SLN imaging.

**Results:**

In this study, TMVP1-modified polymer nanomaterials were loaded with the near-infrared (NIR) fluorescent dye, indocyanine green (ICG), to prepare a molecular imaging TMVP1-ICG nanoparticles (NPs) to identify tumor metastasis in SLN at molecular level. TMVP1-ICG-NPs were successfully prepared using the nano-precipitation method. The particle diameter, morphology, drug encapsulation efficiency, UV absorption spectrum, cytotoxicity, safety, and pharmacokinetic properties were determined. The TMVP1-ICG-NPs had a diameter of approximately 130 nm and an ICG loading rate of 70%. *In vitro* cell experiments and *in vivo* mouse experiments confirmed that TMVP1-ICG-NPs have good targeting ability to tumors *in situ* and to SLN with tumor metastasis by binding to VEGFR-3. Effective photothermal therapy (PTT) with TMVP1-ICG-NPs was confirmed *in vitro* and *in vivo*. As expected, TMVP1-ICG-NPs improved ICG blood stability, targeted tumor metastasis to SLN, and enhanced PTT/photodynamic (PDT) therapy, without obvious cytotoxicity, making it a promising theranostic nanomedicine.

**Conclusion:**

TMVP1-ICG-NPs identified SLN with tumor metastasis and were used to perform imaging-guided PTT, which makes it a promising strategy for providing real-time NIR fluorescence imaging and intraoperative PTT for patients with SLN metastasis.

**Supplementary Information:**

The online version contains supplementary material available at 10.1186/s12951-023-01883-6.

## Introduction

Malignant tumors pose a major threat to human life and health. There were 19.3 million new cancer cases and 10.0 million associated deaths worldwide in 2020 [1]. Recurrence and metastasis are the primary causes of cancer-related death [2]. The liver, lungs, bone, and brain are the most common sites of metastasis; however, many cancers, such as malignant melanoma, gastric cancer, and endometrial cancer, favor lymphatic metastasis when malignant metastasis occurs [2–4]. Surgical treatment of many cancers usually involves regional lymph node dissection, which can cause complications, the most common of which is lymphedema [5]. Furthermore, many patients without lymphatic metastasis may undergo this operation and experience serious complications [6]. A sentinel lymph node is defined as the first lymph node to receive lymphatic drainage from the original tumor [7, 8]. This is the first stage of tumor lymphatic metastasis and is closely associated with the patient’s prognosis. Sentinel lymph node biopsy has become part of the standard of treatment for breast cancer and melanoma [9, 10]. The accurate identification of SLN with metastases is essential for the implementation of effective personalized surgical programs.

Traditional methods used for the detection of SLN, such as computed tomography (CT) and magnetic resonance imaging (MRI), are mainly at the anatomical level and have considerable false-negative and false-positive rates [11]. Radioactive detection methods, such as ^99^Tm-labeled radioactive substances, are also used to image SLN. However, their low resolution and inconvenient operation hinder their further usage [12]. Recently, intraoperative real-time SLN imaging has been performed for a variety of cancers, such as endometrial cancer, vulvar cancer, breast cancer, skin malignant melanoma, and gastric cancer [13–19]. However, currently used dyes that rely solely on the lymphatic drainage pathway have low specificity and weak penetration, making it difficult to image the internal tissues of the body [20]. Notably, NIR fluorescence dyes have stronger tissue penetration capabilities and higher signal-to-noise ratios [11]. Indocyanine Green (ICG) is a NIR fluorescent dye approved by the U.S. Food and Drug Administration (FDA) and is widely used in the clinical imaging of lymph nodes [21–23]. Although it is also a safe photosensitizer, it has obvious shortcomings, such as poor optical stability in aqueous solution, rapid transfer through the SLN, and entry into the secondary draining lymph node, which limit its clinical application [24].

Molecular imaging relying on the high-affinity binding of ligands to receptors may enable the detection and characterization of diseases at an early stage and real-time monitoring of treatment responses [25]. Although monoclonal antibodies have good affinity, their longer blood circulation time leads to a high background signal. As a macromolecule, it is difficult for an antibody to penetrate solid tumor sites [26, 27], and its high cost makes it unavailable. Peptides have the advantages of low molecular weight, diversity, low immunogenicity, and ease of synthesis and chemical modification, making them ideal ligands for molecular-targeting coupling probes [20]. Currently, there are a variety of peptide-modified drugs targeting integrins, selectin and folate, which have been effective at the imaging and treatment of melanoma, ovarian cancer, and pancreatic cancer [28, 29]. Two tumor-targeting peptides, TMTP1 and TMVP1 [30] were developed in our laboratory. TMTP1 targets tumors *in situ* and metastases by binding to XPNPEP2, whereas TMVP1 is better for lymph node imaging as it binds to vascular endothelial growth factor receptor-3 (VEGFR-3) [31–36]. TMVP1 has a higher affinity and more rapid tumor uptake than other VEGFR-3-targeting agents, such as monoclonal antibodies or peptides [37].

In recent years, the emergence of functional nanomaterials has provided new ideas for improving the detection sensitivity and specificity of imaging agents [38–40]. Nanoimaging agents can utilize enhanced permeability and retention effects (EPR) to promote the accumulation of imaging agents, reduce nonspecific distribution, and improve imaging specificity [41, 42]. Nanoparticle-encapsulated dyes exhibit stronger fluorescence and better photostability than free dyes [43]. Dyes carried by nanomaterials not only enhance tumor targeting through the EPR effect, but can also be coupled with tumor-targeting ligands on nanoparticles for molecular imaging [44]. At present, most nanoprobes target the primary site of the tumor, whereas few target SLN with tumor metastasis.

VEGFR-3 is a receptor for VEGF-C/-D and it regulates vascular remodeling in tumor-metastatic lymph nodes [4, 45]. It is a key molecule that promotes lymphangiogenesis in various cancers, including breast, ovarian, lung, and colon cancers [46–48]. TMVP1, a five-amino peptide with the core sequence of LARGR, end-collateral amide bond into a ring, and with one cysteine residues at the end was screened for VEGFR-3 binding using bacterial flagellin library display technology in our laboratory. In our previous study, a new type of VEGFR-3 molecular imaging agent, Ga^68^-DOTA-TMVP1, prepared by coupling TMVP1 with radioactive Ga^68^, was successfully used in preclinical experiments and achieved good imaging results in patients with malignant gynecological tumors [37]. In this study, we designed and synthesized a TMVP1-modified ICG nanoprobe, TMVP1-ICG-NP, using poly (ethylene glycol)-poly (lactic-co-glycolic acid) (PEG-PLGA), a promising biodegradable polymer. The nanoprobe characteristics such as stability, size, charge, and cellular uptake, were detected *in vitro*, and its targeted tracing ability for SLN with tumor metastasis was verified *in vivo*. Finally, photothermal therapy (PTT) and photodynamic therapy (PDT) using TMVP1-ICG-NP were confirmed *in vitro* and *in vivo*.

**Scheme 1:**
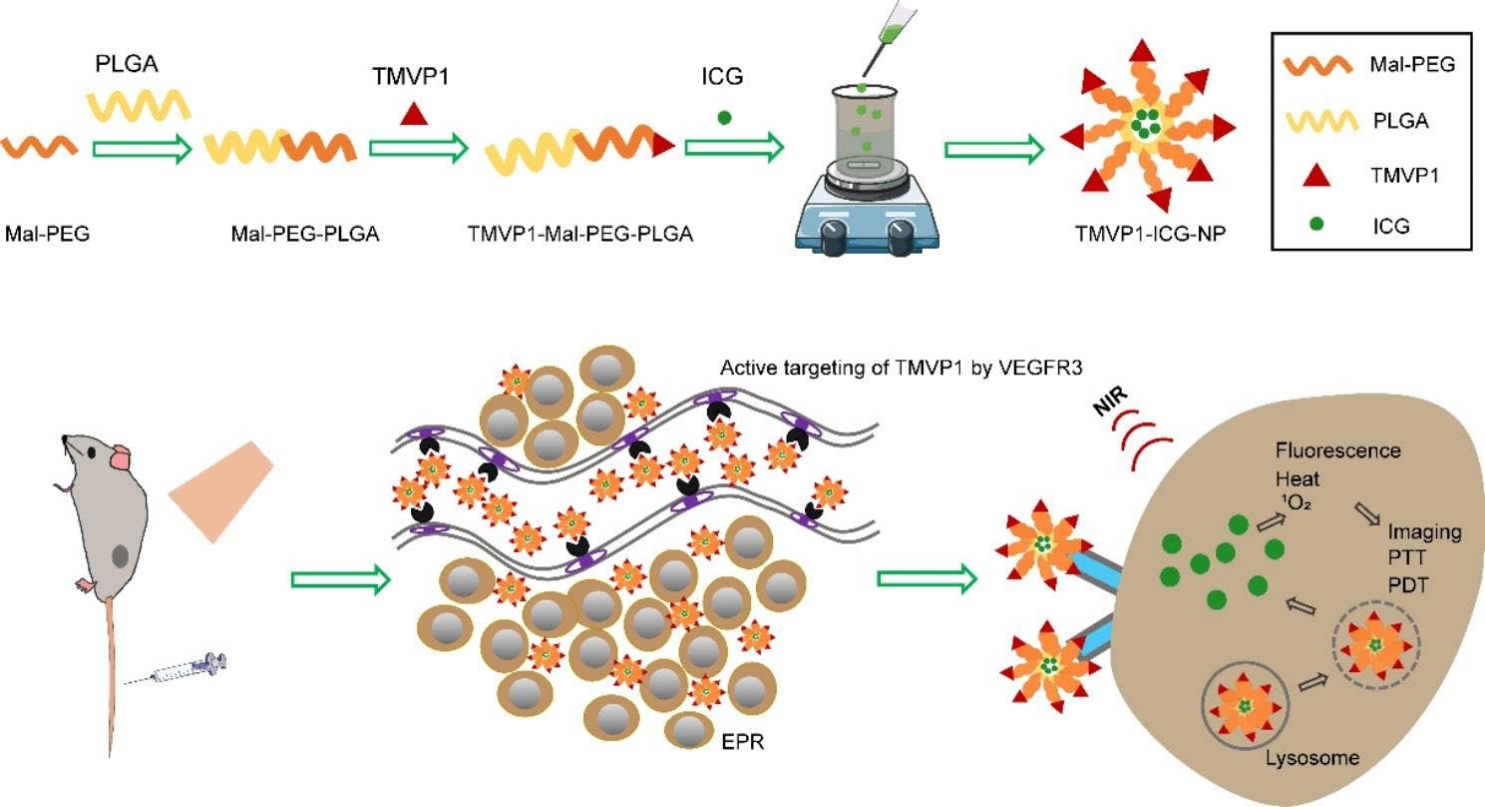
Schematic illustration of the preparation of TMVP1-ICG-NPs and targeted imaging and PTT/PDT based on TMVP1-ICG-NP. Maleimide bond-terminated PEG (Mal-PEG) was chemically linked to PLGA to obtain Mal-PEG-PLGA, and peptide TMVP1 was connected to PEG-PLGA by reaction with the maleimide bond of PEG. Next, TMVP1-ICG-NPs were prepared by the nano-precipitation under magnetic stirring. Self-assembly occurred through hydrophobic forces of PLGA and the ICG was wrapped into the hydrophobic shell, while PEG, as a hydrophilic branch of the shell, enabled the nanoprobe to dissolve in water. The nanoprobe was actively targeted to new lymphatic vessels with tumor metastasis by binding to VEGFR-3 after tail vein injection. ICG was released by lysosome degradation, and then imaging, PTT, and PDT were performed under the irradiation of NIR.

## Results

### Preparation and characterization of ICG-loaded TMVP1-PEG-PLGA micelles

**Fig. 1:**
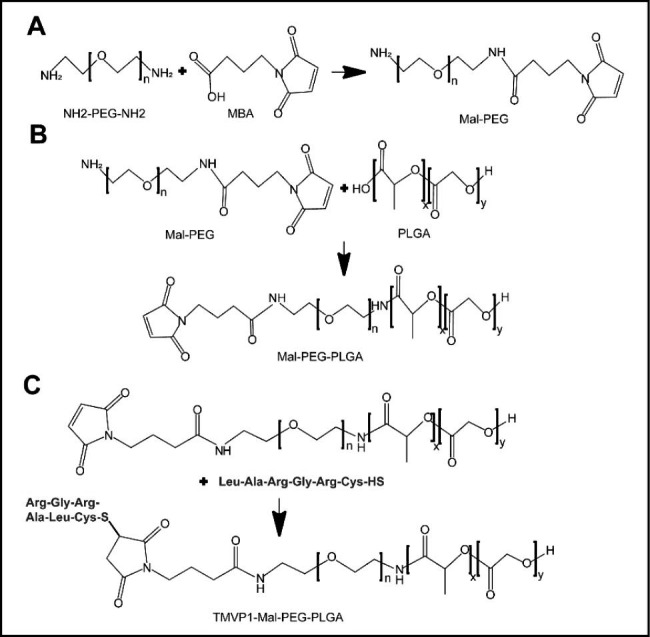
Synthesis of TMVP1-modified PEG-PLGA. (A) The synthetic of Mal-PEG. (B) The synthetic of Mal-PEG-PLGA. (C) The synthetic of TMVP1-Mal-PEG-PLGA.

**Fig. 2:**
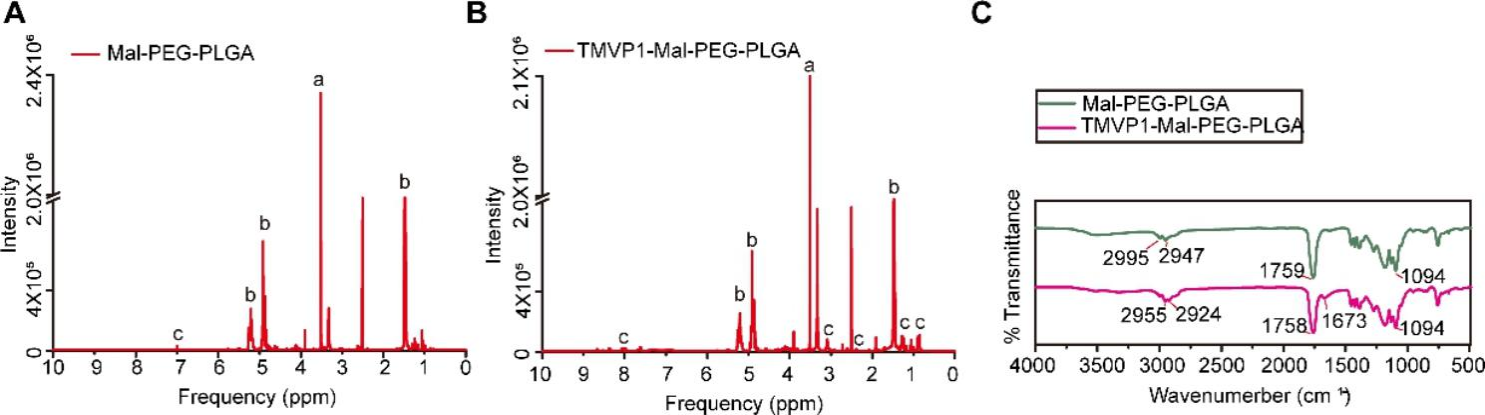
^1^HNMR and FT-IR of nanomaterials. (A) ^1^HNMR spectra of Mal-PEG-PLGA. (B) ^1^HNMR spectra of TMVP1-Mal-PEG-PLGA. (C) FT-IR spectra of Mal-PEG-PLGA and TMVP1-Mal-PEG-PLGA.

TMVP1-PEG-PLGA was synthesized using thiol-maleimide click chemistry. First, the amino-terminus of PEG was chemically combined with 4-Maleimidobutyric Acid (MBA) to form a maleimide bond (Fig. 1A). Next, Mal-PEG was allowed to react with PLGA (Fig. 1B). Finally, TMVP1 was linked to PEG-PLGA via the maleimide bond (Fig. 1C). The connection between the polypeptide TMVP1 and the PEG-PLGA polymer material was verified. As shown in Fig. 2A, the proton nuclear magnetic resonance (^1^ H-NMR) results suggested that the -CH_2_ peak of PEG in Mal-PEG-PLGA appeared at 3.51 ppm (a), the -CH_3_, -CH_2_, and -CH of PLGA [49] appeared at 1.48 ppm, 4.92 ppm, and 5.21 ppm (b), and the peak at 7.00 ppm (c) was represent the maleimide bond [31]. Figure 2B showed the ^1^H-NMR result of TMVP1-Mal-PEG-PLGA, similarly, the -CH_2_ peak of PEG appeared at 3.51 ppm (a), the -CH_3_, -CH_2_, and -CH of PLGA appeared at 1.48 ppm, 4.92 ppm, and 5.21 ppm (b). Along with the disappearance of the maleimide bond peak, we observed the emergence of the polypeptide cTMVP1 peaks at 7.61–8.67 ppm, 3.19 ppm, 2.37ppm, 1.24–1.27 ppm, and 0.85–0.90 ppm (c). For the FT-IR analysis (Fig. 2C), characteristic absorbed bonds of 2995 − 2947 cm^− 1^ and 2955 − 2924 cm^− 1^ belonged to the stretching and bending vibration of -CH_3_ and -CH_2_, the peaks at 1759 cm^− 1^ and 1758 cm^− 1^ attributed to the stretching vibration of carbonyl group -C = O, and the peaks at 1094 cm^− 1^ attributed to C-O stretching. In the spectrum of TMVP1-Mal-PEG-PLGA, the appearance of 1673 cm^− 1^ absorption peak belonged to the -CO-NH- of polypeptide cTMVP1. These results indicated the successful synthesis of TMVP1-Mal-PEG-PLGA. The verification results of other intermediates products in the synthesis reaction are shown in Figure S3.

**Fig. 3:**
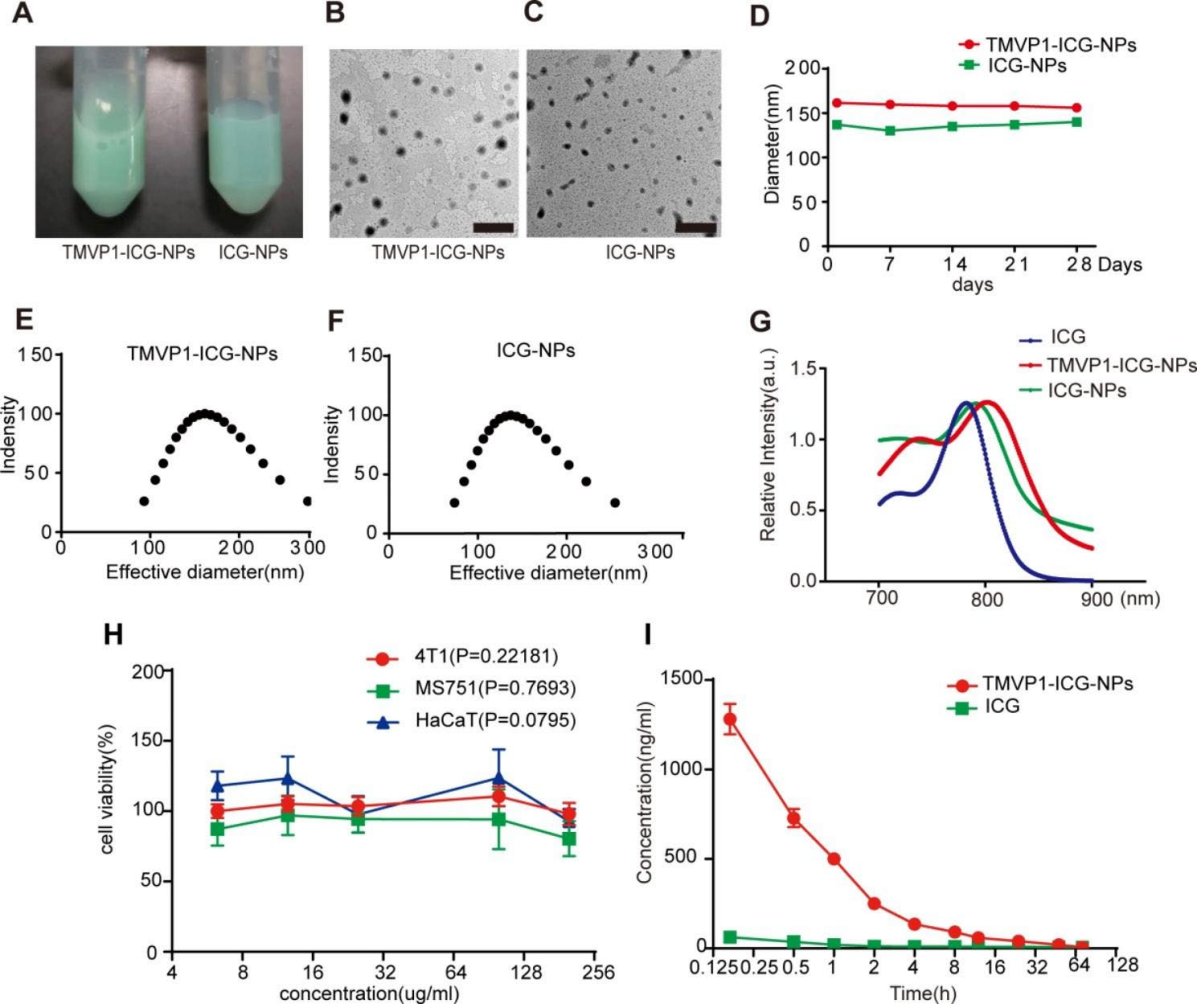
The characteristics of TMVP1-ICG-NPs. (A) The appearance of TMVP1-ICG-NPs and the control ICG-NPs. (B-C) TEM images of TMVP1-ICG-NPs and ICG-NPs (scale bar 500 nm). (D) Size stability of TMVP1-ICG-NPs and ICG-NPs stored in phosphate-buffered saline (PBS, pH 7.4) at 4℃. (E-F) The size distribution of TMVP1-ICG-NPs and ICG-NPs was determined by DLS. (G) UV/Vis spectra of free ICG, ICG-NPs, and TMVP1-ICG-NPs in PBS. (H) Cytotoxicity of different ICG concentrations (6.25, 12.5, 25, 100, and 200 µg /mL) in TMVP-ICG-NPs in 4T1, MS751, and HaCaT cells after 24 h of incubation. (I) Blood metabolism characteristics of TMVP1-ICG-NPs and free ICG in SD rats via tail vein injection of 500 µg of equivalent ICG. Values of *p* < 0.05 were considered significant (ns: no significance, *p < 0.05, **p < 0.01, ***p < 0.001, ****p < 0.0001)

The TMVP1-ICG-NPs and the control nanoprobe, ICG-NPs, were prepared by the nano-precipitation method, and had a green appearance (Fig. 3A). Transmission electron microscopy (TEM) images showed that TMVP1-ICG-NPs and ICG-NPs had a uniform spherical appearance, with average diameters of 130 nm and 110 nm, respectively (Fig. 3B and C). The average hydrodynamic diameters and surface charges of the TMVP1-ICG-NPs and ICG-NPs, detected by dynamic light scattering (DLS) (PBS, PH7.4), are shown in Table 1. Both nanoprobes exhibited good particle size stability over 28 d (Fig. 3D). TMVP1-ICG-NPs and ICG-NPs had similar average diameters of approximately 150 and 130 nm, respectively, with narrow distributions (Fig. 3E and F). The zeta potentials of the TMVP1-ICG-NPs and ICG-NPs were between − 8.12 mV and − 5.21 mV, which indicated that the particles could retain sufficient stability in an aqueous dispersion and maintain sufficient colloidal stability. The ICG encapsulation efficiency of the TMVP1-ICG-NPs was 70.4% and the ICG encapsulation efficiency of the ICG-NPs was 63.7%. Compared to free ICG, the peak absorbances of TMVP1-ICG-NPs and ICG-NP shifted from 780 to 810 nm (Fig. 3G), which was consistent with the results reported in the literature [50, 51]. This is because the hydrophobic environment in which the ICG molecules resided reduced the fluorescence quenching caused by the concentration-dependent self-aggregation of free ICG exposed to water [52, 53]. Meanwhile, ICG monomers in the nanoparticles were polymerized to a certain extent caused the shifted of the peak, and the maximum absorption wavelength of ICG in TMVP1-ICG-NPs more shifted than the maximum absorption wavelength of ICG in ICG-NPs, possibly due to the fact that TMVP1-ICG-NPs had a higher ICG loading [54].

4T1, MS751, and HaCaT cells were used to perform Cell Counting Kit-8 (CCK8) assays to verify the cytotoxic effect of TMVP1-ICG-NPs at a maximum concentration of 200 µg/mL of ICG. The higher concentrations of the nanoprobe had no obvious toxic effects on cells *in vitro*, with a cell survival rate close to 90% at a 200 µg/mL ICG equivalents (Fig. 3H), which suggested that the nanoprobe was safe and non-toxic. Pharmacokinetic analysis performed in Sprague–Dawley (SD) rats showed that the ICG metabolism rate was slower for TMVP1-ICG-NPs than free ICG. Free ICG in the blood circulation was almost completely degraded within 10 min, while the ICG content in TMVP1-ICG-NPs was 20.2 times than the free ICG content at 10 min. ICG in TMVP1-ICG-NPs was gradually metabolized over time, but was still 2.5 times higher than the free ICG content at 48 h (Fig. 3I), suggesting that the nanomicelles delayed the degradation of ICG in the blood.

**Table 1:** The basic characteristics of NPs

Nanoparticles	Diameter(nm)	Zeta potential(mV)	EE (%)	Polydispersity
TMVP1-ICG-NPs	156.4 ± 3.15	-8.12 ± 0.63	70.4 ± 0.35	0.095 ± 0.019
ICG-NPs	130.2 ± 2.10	-5.21 ± 0.23	63.7 ± 0.08	0.099 ± 0.031

### ***In vitro*** cellular uptake

**Fig. 4:**
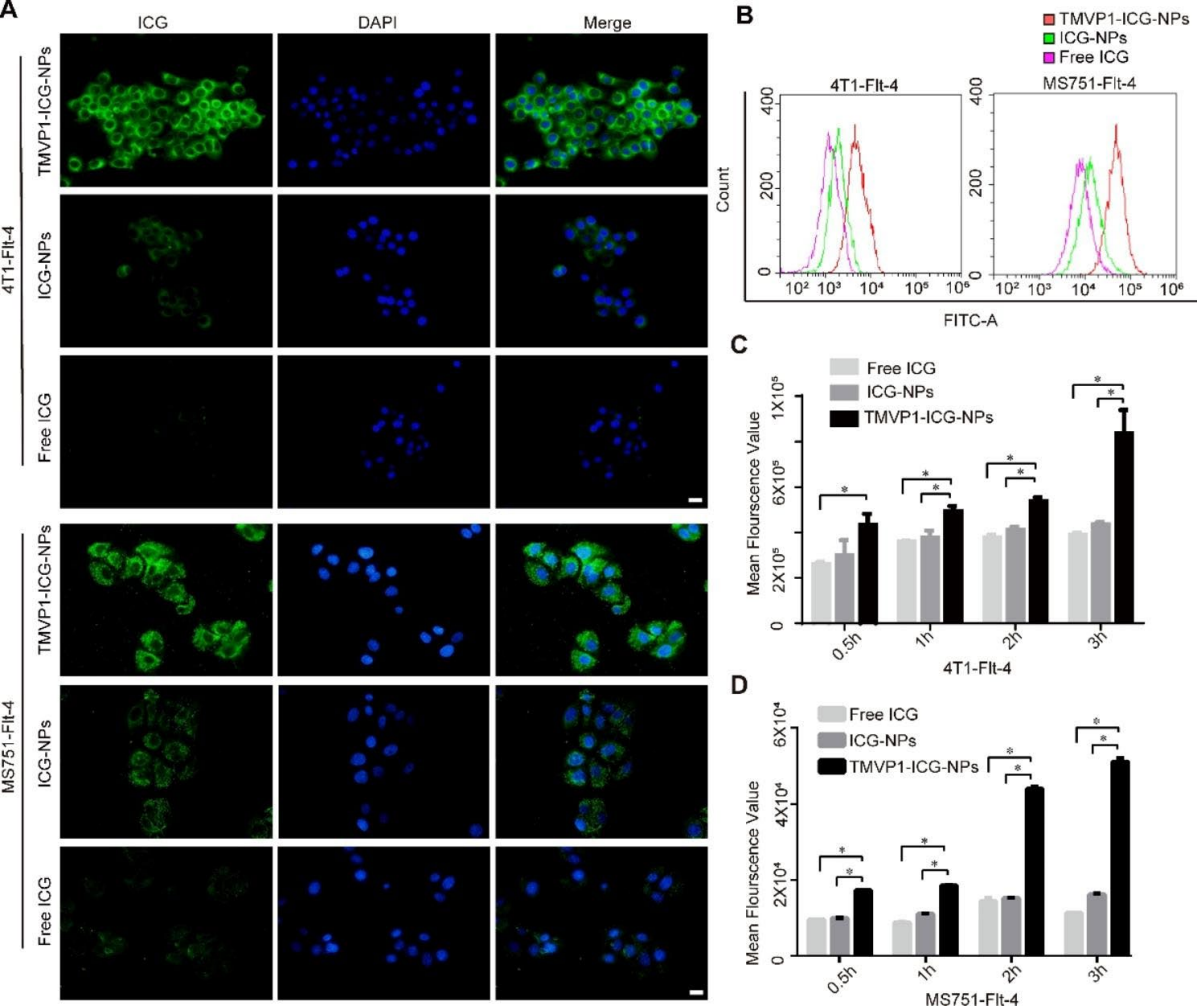
*In vitro* cell targeting ability of TMVP1-ICG-NPs. (A) Confocal fluorescence images of cells treated with TMVP1-ICG-NPs, ICG-NPs, and free ICG (ICG concentration = 100 ng/mL) after 3 h of incubation (scale bar, 20 μm). The fluorescence images of 4’,6-diamidino-2-phenylindole (DAPI) and ICG were obtained at excitation wavelengths of 405 and 633 nm. 4T1 and MS751 cells overexpressing VEGFR-3 were generated via a lentiviral vector, and called 4T1-Flt-4 and MS751-Flt-4 cells, respectively. (B) TMVP1-ICG-NPs, ICG-NPs, and free ICG uptake by 4T1-Flt-4 and MS751-Flt-4 cells were detected by flow cytometry after 3 h of incubation. (C-D) The mean fluorescence intensity of 4T1-Flt-4 and MS751-Flt-4 cells treated with TMVP1-ICG-NPs, ICG-NPs, and free ICG (ICG concentration = 100 ng/mL) at 0.5 h, 1 h, 2 h, and 3 h detected by flow cytometry. Values of *p* < 0.05 were considered significant (ns: no significance, *p < 0.05, **p < 0.01, ***p < 0.001, ****p < 0.0001)

VEGFR-3-overexpressing cell lines, 4T1-Flt4 and MS751-Flt4, were successfully generated by lentiviral transfection. Western blotting confirmed that the VEGFR-3-overexpressing cell lines had higher VEGFR-3 expression levels than control 4T1 and MS751 cells (Figure S4). The uptake of TMVP1-ICG-NPs, ICG-NPs, and free ICG by tumor cells was detected by fluorescence microscopy, and it was observed that the cells had significantly increased uptake of TMVP1-ICG-NPs compared with ICG-NPs and free ICG (Fig. 4A). Figure 4B showed drug uptake by 4T1-Flt-4 and MS751-Flt-4 cells, as detected by flow cytometry after 3 h of incubation. The results were consistent with those obtained by immunofluorescence. The results of flow cytometry (Fig. 4C and D) showed that the uptake of TMVP1-ICG-NPs by the two cell lines increased with increasing incubation time, and the differences between the groups were statistically significant (*p* < 0.05). ICG-NPs and free ICG uptake almost reached the maximum level within 0.5 h, and was significantly less than TMVP1-ICG-NPs uptake over time. This suggests that ICG-NPs and free ICG enter cells through passive uptake, and the uptake of drugs by cells is significantly slowed when the extracellular and intracellular drug concentration differences decrease. In contrast, TMVP1-ICG-NPs had greater uptake as they bind to VEGFR-3 expressed on the cell membrane and enter the cells by active intake.

### Dynamics and bio-distribution of TMVP1-ICG-NPs

**Fig. 5:**
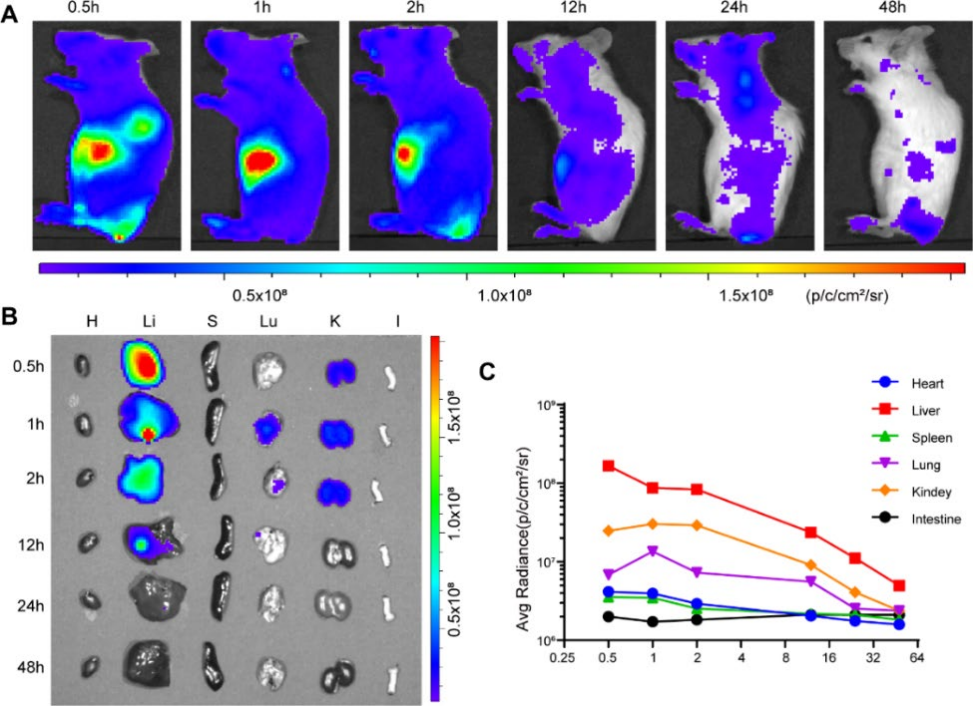
Dynamics and bio-distribution of TMVP1-ICG-NPs. (A) Near infrared (NIR) fluorescence images of normal BALB/c mice at different times (0.5 h, 1 h, 2 h, 12 h, 24 h, 48 h) after the injection of TMVP1-ICG-NPs at a dose of 1.0 mg/kg. (B) Representative NIR fluorescence images of the main dissected organs at different time points after injection (H, heart; Li, liver; S, spleen; Lu, lung; K, kidney; I, intestine). (C) The mean NIR fluorescence signal intensity of the isolated organs was measured using the ROI tool.

To evaluate the metabolism of TMVP1-ICG-NPs *in vivo*, TMVP1-ICG-NPs were intravenously injected into normal BALB/c mice and monitored for 48 h. The results showed that TMVP1-ICG-NPs were dispersed throughout the body at 0.5 h post-injection, followed by accumulation mainly in the liver (Fig. 5A). The overall fluorescence signal decreased with time and metabolism mainly occurred in the liver, kidneys, and lungs (Fig. 5B). The time profiles of the mean fluorescence intensities of the organs are depicted in Fig. 5C. These results suggested that the nanoprobe was cleared through the liver and kidney pathways and the fluorescence intensity reached its highest level 1 h after the injection which was consistent with our previous research [31, 35]. Most of the nanoprobes were cleared 48 h after administration, which is considered safe *in vivo*.

### Subcutaneous tumor imaging ***in vivo***

**Fig. 6:**
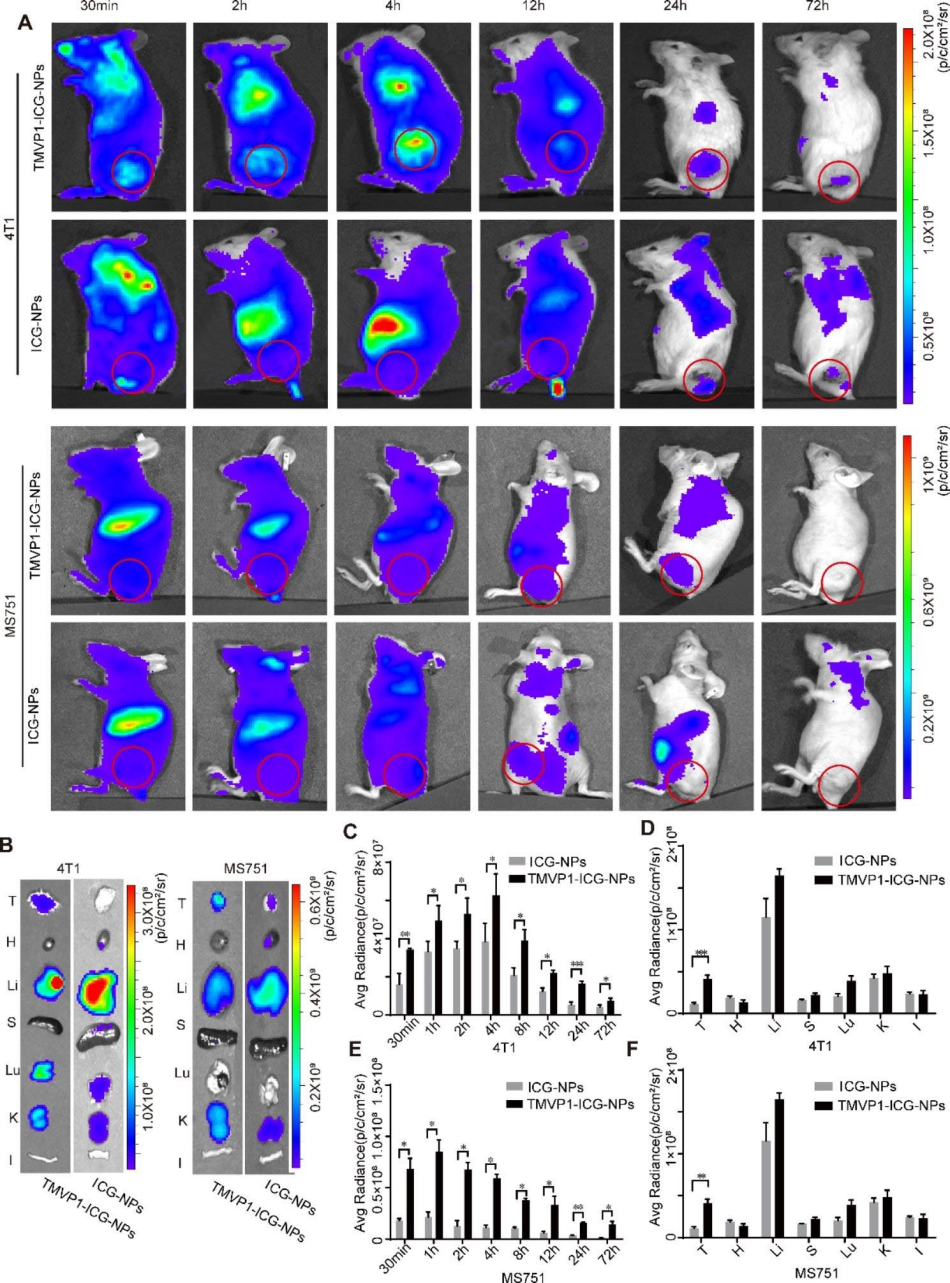
Subcutaneous tumor imaging *in vivo*. (A) NIR fluorescence images of subcutaneous 4T1 and MS751 xenograft model mice at different time intervals post-injection (0.5 h, 2 h, 4 h, 12 h, 24 h, 72 h). TMVP1-ICG-NPs and ICG-NPs were injected via the tail vein at a dose of 1.0 mg/kg. The red circle showed the tumor site with fluorescence. (B) Representative NIR fluorescence images of the isolated tumors and organs at 24 h post-injection (T, tumor; H, heart; Li, liver; S, spleen; Lu, lung; K, kidney; I, intestine). (C) The mean NIR fluorescence signal intensity of 4T1 subcutaneous tumors was measured using the ROI tool at different time points in the two groups. (D) The mean NIR fluorescence signal intensity of the 4T1 subcutaneous tumor and organs from mice of the two groups at 24 h. (E) The mean NIR fluorescence signal intensity of MS751 subcutaneous tumors was measured using the ROI tool at different time points in the two groups. (F) The mean NIR fluorescence signal intensity of the MS751 subcutaneous tumor and organs from mice of the two groups at 24 h. Values of *p* < 0.05 were considered significant (ns: no significance, *p < 0.05, **p < 0.01, ***p < 0.001, ****p < 0.0001)

4T1 and MS751 tumor-bearing mice were used to investigate the tumor-targeting ability of TMVP1-ICG-NPs. Compared with the control ICG-NPs group, the TMVP1-ICG-NPs group showed more nanoprobe aggregation at the tumor site (Fig. 6A and B). Statistical analysis of the near-infrared fluorescence intensity values of tumor sites in mice with 4T1 subcutaneous tumors and MS751 subcutaneous tumors showed a statistically significant difference in the aggregation of TMVP1-ICG-NPs and ICG-NPs at the tumor sites 30 min after administration. The difference between TMVP1-ICG-NPs and ICG-NPs aggregated at the tumor site was statistically significant at 1, 2, 4, 8, and 12 h, and the difference between the two groups was the largest after 24 h and could be sustained up to 72 h (Fig. 6C and E). Statistical analysis was performed on the NIR fluorescence images of the dissected tumors and major organs, such as the heart, liver, spleen, lung, kidney, and intestine. The results showed statistically significant differences in NIR fluorescence intensity at the tumor sites (4T1 group, *p* = 0.00054; MS751 group, *p* = 0.00120), but no significant differences for the other major organs of both 4T1 and MS751 groups (Fig. 6D and F). These results indicated that intravenously injected TMVP1-ICG-NPs specifically target tumor sites *in vivo*, suggested the active targeting of TMVP1-ICG-NP compared to local injection model [55].

### Optical imaging of tumor metastatic SLN with popliteal lymph node metastasis

**Fig. 7:**
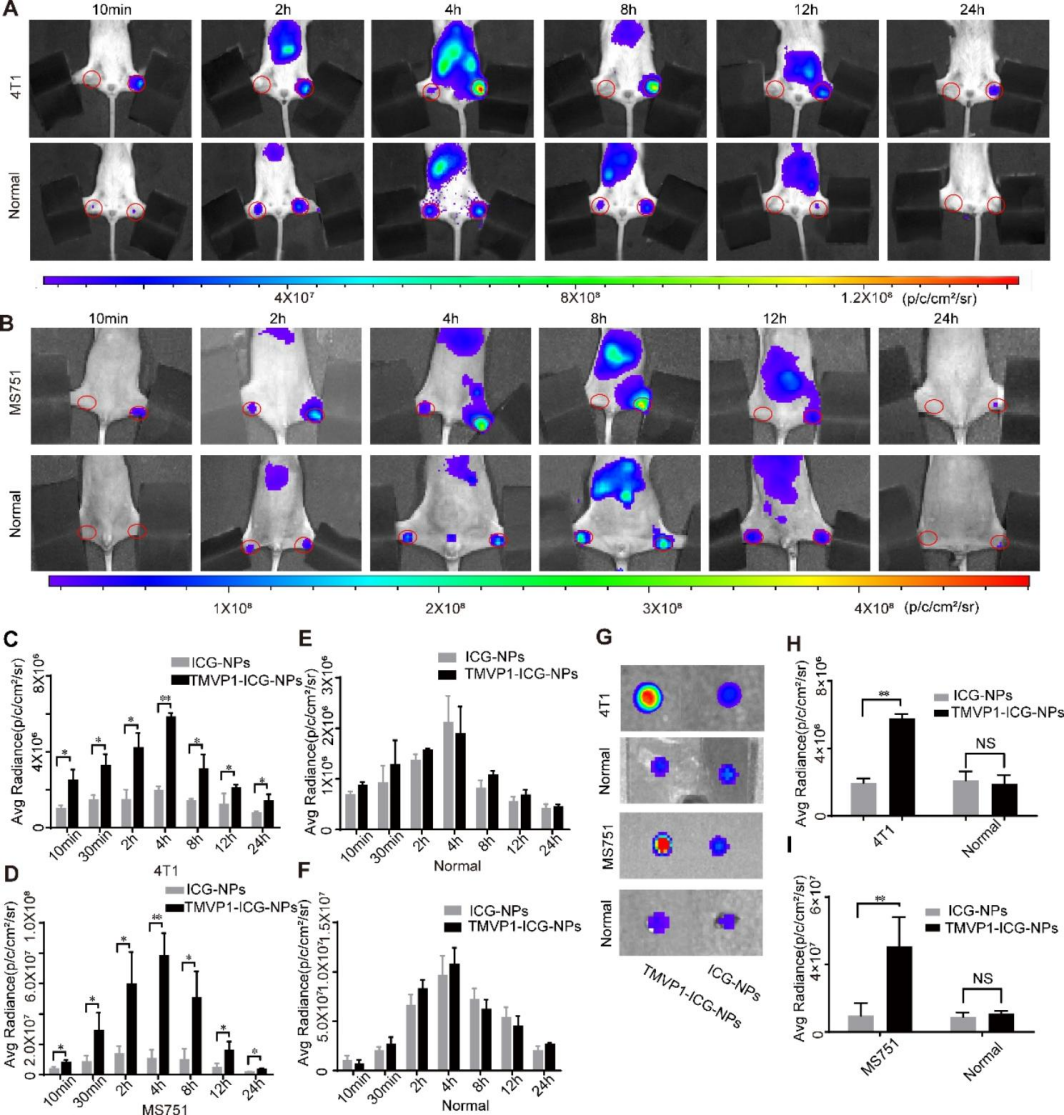
Lymph node metastasis foci imaging *in vivo*. (A-B) NIR fluorescence images of 4T1/MS751 footpad lymph node metastasis model mice and normal mice at different time points (10 min, 2 h, 4 h, 8 h, 12 h, and 24 h). The right footpad was injected with TMVP1-ICG-NPs and the left footpad was injected with ICG-NPs. (C-F) The mean NIR fluorescence signal intensity of the lymph nodes with tumor metastasis and normal lymph nodes at the different time points after bilateral injection of the corresponding drugs. (G) Representative NIR fluorescence images of isolated lymph nodes with tumor metastasis and normal lymph nodes at 4 h post-injection. (H-I) The mean NIR fluorescence signal intensity of popliteal lymph nodes of (G) was measured using the ROI tool. Values of *p* < 0.05 were considered significant ns: no significance, *p < 0.05, **p < 0.01, ***p < 0.001, ****p < 0.0001)

We used a mouse popliteal lymph node metastasis model as a SLN metastasis model to verify the ability of TMVP1-ICG-NPs to target tumor metastasis SLN. We used dual popliteal lymph node metastases model to avoid individual differences of mouse. In Balb/c and Balb/c-Null mice with popliteal lymph node metastases, the right side injected with TMVP1-ICG-NPs showed stronger ICG NIR fluorescence than the left side injected with ICG-NPs for 10 min (Fig. 7A and B), indicated that TMVP1-ICG-NPs has more retention at tumor metastasis SLN. Meanwhile, normal Balb/c mice and Balb/c-Null mice had almost the same NIR fluorescence intensity of ICG in the right and left popliteal fossa after administration (Fig. 7A and B), which suggests that the drainage process of the two nanoprobes in the lymphatic pathway of the lower limbs of normal mice was similar. We used the ROI tool measure the NIR fluorescence intensities at popliteal lymph node metastasis position of both 4T1 and MS751 group. The difference between the left and right sides was statistically significant at different time points, reaching a maximum difference at 4 h (Fig. 7C and D) for popliteal lymph node metastasis group of both 4T1 and MS751. Different from previous studies, the maximum fluorescence intensity value caused by polypeptide-mediated active targeting appeared later than that of EPR [56]. Statistical analysis suggested no statistically significance (Fig. 7E and F) between the right and left sides NIR fluorescence intensity at normal Balb/c and Balb/c-Null mice. The popliteal lymph nodes were dissected 4 h after administration and it could be clearly seen that the intensity of the TMVP1-ICG-NPs was stronger than the intensity of the ICG-NPs (Fig. 7G). The difference between the NIR fluorescence intensities of the TMVP1-ICG-NPs and ICG-NPs sides was statistically significant (4T1 group, *P* = 0.0012; MS751 group, *P* = 0.0020; Fig. 7H and I) of both 4T1 and MS751 group. In comparison, the NIR fluorescence intensities of the TMVP1-ICG-NPs and ICG-NPs in the bilateral popliteal lymph nodes of normal Balb/c and Balb/c-Null mice were similar (Fig. 7G), without statistically significance (Fig. 7H and I). Overall, TMVP1-ICG-NPs specifically recognized SLN with tumor metastasis in a mouse tumor lymph node metastasis model.

We subsequently performed immunohistochemistry to detect the expression of VEGFR-3 in popliteal lymph nodes with tumor metastasis. These results suggested that VEGFR-3 was expressed in popliteal lymph nodes during tumor metastasis (Fig. 10A). The yellow arrows showed the expression region of VEGFR-3, and the expression of VEGFR-3 has a tubular distribution, similar to the course of the new lymphatic vessels. The immunohistochemistry results suggest that in lymph nodes with tumor metastasis, new lymphatic vessels express VEGFR-3. This suggests that TMVP1-ICG-NPs had greater aggregation and longer retention in SLN with tumor metastasis by binding to VEGFR-3.

### PTT and PDT effects of TMVP1-ICG-NPs

**Fig. 8:**
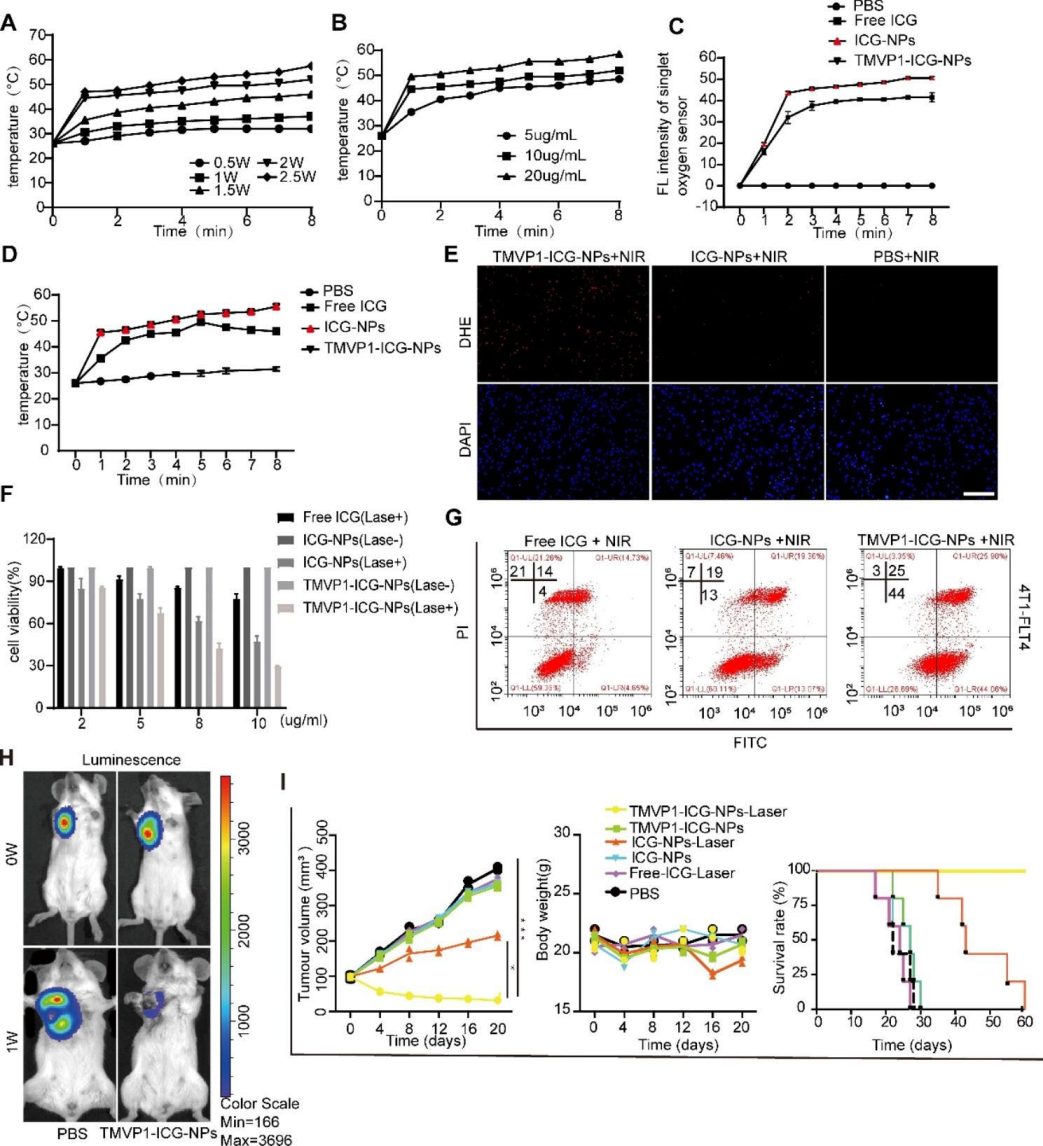
PTT and PDT using TMVP1-ICG-NPs. (A) The photothermal effect of TMVP1-ICG-NPs at different laser powers. (B) The photothermal effect of TMVP1-ICG-NPs at different ICG concentrations. (C) FL intensity of a single oxygen sensor. (D) Differences in the photothermal effect between PBS, free ICG, ICG-NPs, and TMVP1-ICG-NPs. (E) Fluorescence images of cells treated with TMVP1-ICG-NPs, ICG-NPs, and free ICG after irradiation (scale bar, 200 μm). The cells were treated with 200 µL of dihydroethidium (DHE) solution (5 µM in PBS) to induce ROS production. Fluorescence images of DAPI and 2,7-dichlorofluorescin were obtained at excitation wavelengths of 405 and 529 nm, respectively. (F) Cell viability after PTT and PDT was determined by CCK8 assay. (G) Apoptosis caused by free ICG, ICG-NPs, and TMVP1-ICG-NPs after NIR laser, as detected by flow cytometry. (H) Luminescence images of 4T1-tumor-bearing mice before and after PTT and PDT. (I) Tumor growth curve and mouse body weight growth curve in different treatment groups, and survival analysis. Values of *p* < 0.05 were considered significant (ns: no significance, *p < 0.05, **p < 0.01, ***p < 0.001, ****p < 0.0001)

First, the photothermal and photodynamic effects of TMVP-ICG-NPs were evaluated *in vitro*. We evaluated the temperature changes of 10 µg/mL TMVP-ICG-NPs under different NIR irradiation power levels. Figure 8A showed that the temperature range increased with increasing power. When the NIR laser irradiation was greater than 2.0 W/cm^2^, the temperature rapidly increased above 45℃ within 2 min. This temperature can destroy tumor cells *in vivo*. We then used a 2.5 W/cm^2^ NIR laser to irradiate 5 µg/mL, 10 µg/mL, and 20 µg/mL TMVP1-ICG-NPs, and the results suggested that the temperature increased with the concentration of ICG (Fig. 8B). In contrast with phosphate-buffered saline (PBS), the free ICG, ICG-NPs, and TMVP1-ICG-NPs (10 µg/mL equivalent ICG) produced obvious singlet oxygen and showed a significant increase in temperature under NIR laser irradiation (808 nm, 2.5 W/cm^2^). However, free ICG was more easily quenched than the NPs and it led to a slightly smaller effect (Fig. 8C and D). We also used dihydroethidium (DHE) to determine the reactive oxygen species (ROS) content of living cells after 2.5 W/cm^2^ NIR laser irradiation. As shown in Fig. 8E, red fluorescence, indicating ROS content, was significantly higher in the TMVP-ICG-NPs-treated group than the ICG-NPs- and PBS-treated groups. These results suggested TMVP1-ICG-NPs are promising for PTT and PDT applications. CCK8 and flow cytometry results also suggested that TMVP-ICG-NPs caused the highest level of apoptosis under NIR laser irradiation, compared to ICG-NPs and free ICG (Fig. 8F and G).

4T1-tumor-bearing mice were used to evaluate the utility of TMVP1-ICG-NPs for PTT and PDT *in vivo*. The 4T1-tumor-bearing mice were divided into six treatment groups (PBS, free ICG + laser, ICG-NPs, ICG-NPs + laser, TMVP1-ICG-NPs, and TMVP1-ICG-NPs + laser). An obvious inhibition of tumor growth was observed in the TMVP1-ICG-NPs + laser group (Fig. 8H and I), ICG-NPs + laser group caused weaker tumor inhibition than in the TMVP1-ICG-NPs + laser group, whereas the other groups showed rapid tumor growth. The body weights of the mice were not significantly different between the groups, indicating that NIR laser irradiation with TMVP1-ICG-NPs did not induce cytotoxicity in the mice. The TMVP1-ICG-NPs group also showed a significant prolongation of survival compared to the ICG-NPs group and the other groups (Fig. 8I).

### ***In vivo*** toxicity of TMVP1-ICG-NPs

**Fig. 9:**
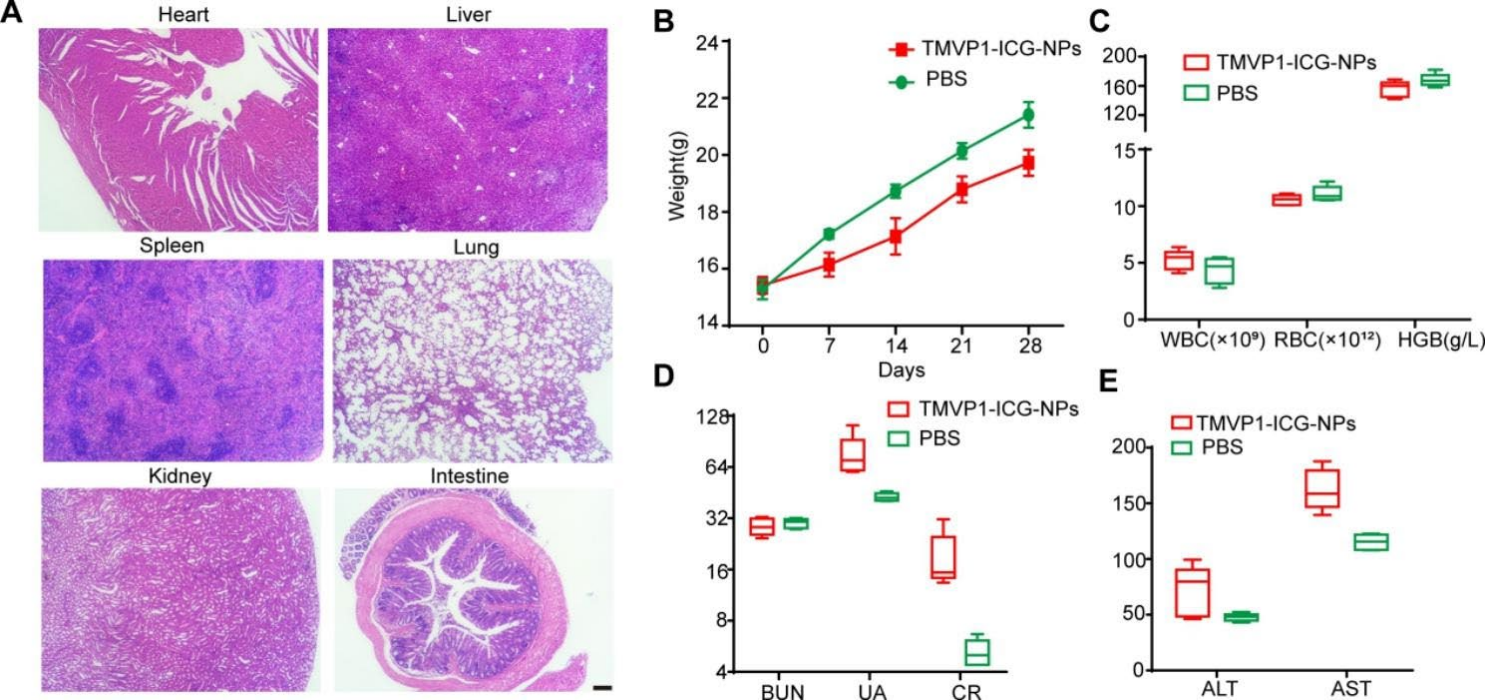
Safety analysis *in vivo*. (A) Hematoxylin and eosin staining images of the organs. (B) Body weight changes in the TMVP1-ICG-NPs and PBS groups in the acute toxicity study. (C) Blood cell analysis including erythrocytes (RBC), leukocytes (WBC), and hemoglobin (HGB) at the endpoint of the observation. (D-E) Biomedical indicators including BUN, UA, CR, ALT, and AST at the endpoint of the observation.

To examine the toxicity of NPs *in vivo*, high doses of TMVP1-ICG-NPs were intravenously injected into healthy mice. There was a slight weight loss over 28 d after drug administration, but the weight of the mice remained within the normal range. The mice were sacrificed 28 d post-injection. Histological analysis of the major organs showed no signs of overt toxicity, such as tissue degeneration or necrosis, compared to the effects of PBS treatment (Fig. 9A). Body weight, blood cell analyses, including erythrocyte, leukocyte, and hemoglobin levels, were normal in the TMVP1-ICG-NPs group. The levels of alanine aminotransferase (ALT), aspartate aminotransferase (AST), blood urea nitrogen (BNU), uric acid (UR), and creatinine (CR) in the TMVP1-ICG-NPs group were within the normal range, although these indicators fluctuated slightly compared to the levels in the PBS group (Fig. 9B-E). These results suggested that ICG-loaded micelles were nontoxic *in vivo* at a dose of 10 mg/kg, which is approximately 10 times higher than the imaging dose of ICG used in most studies. Our results confirmed the nontoxicity and biocompatibility of the ICG-loaded nanoprobes.

### Histopathological examination

**Fig. 10:**
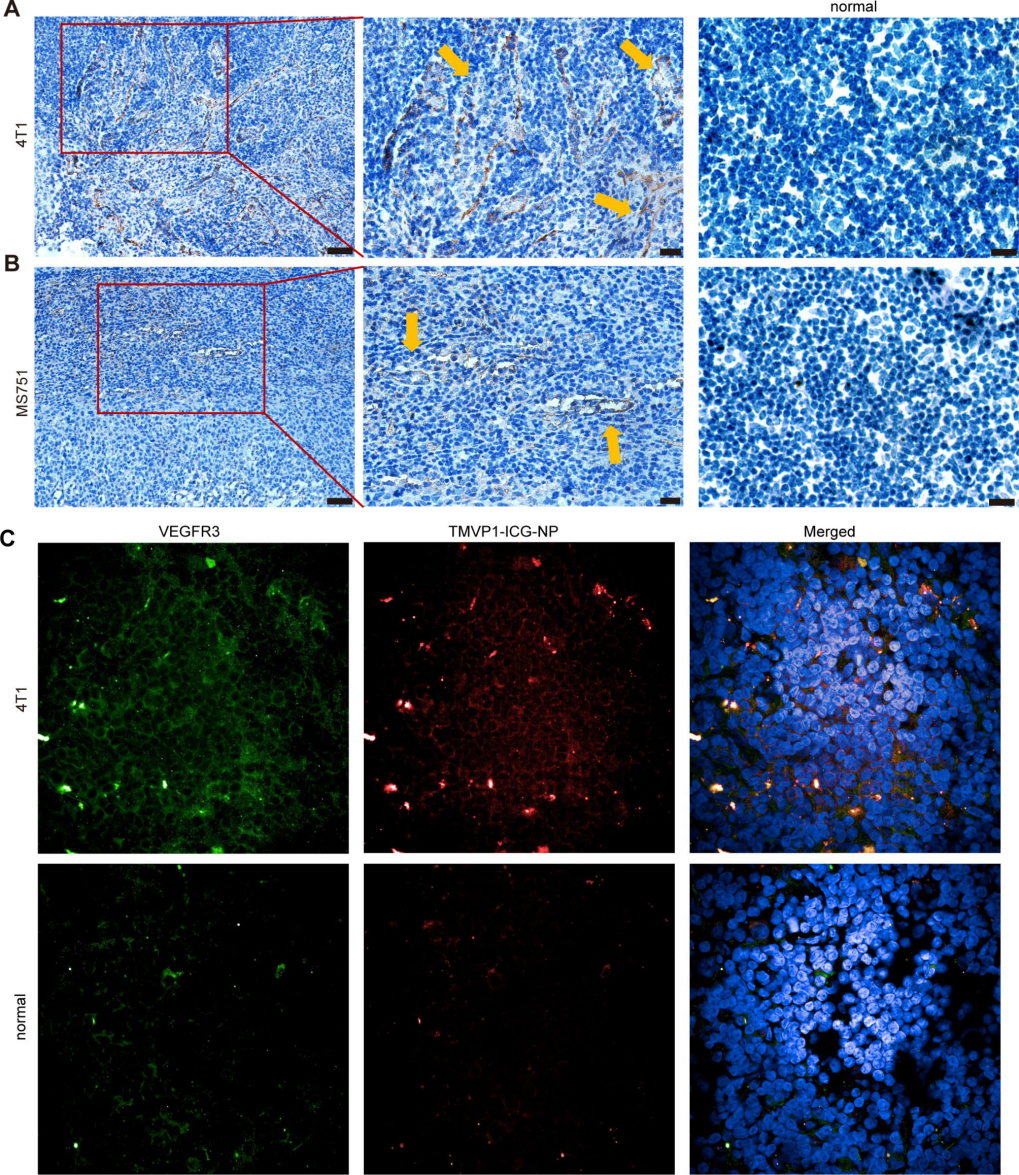
Immunohistochemical examination. (A-B) Immunohistochemical staining showed the expression of VEGFR-3 in lymph nodes with 4T1/MS751 tumor metastases (left scale bar is 50 μm; middle scale bar is 20 μm; right scale bar is 50 μm). (C) Immunofluorescence showed the overlap of VEGFR-3 and ICG in lymph node metastases.

Immunohistochemistry was performed on popliteal lymph nodes with metastases. The results showed that both 4T1 and MS751 popliteal lymph nodes with metastases expressed VEGFR-3, and the distribution was along the new lymphatic vessels (Fig. 10A and B). Immunofluorescence analysis showed that the fluorescence of VEGFR-3 and TMVP1-ICG-NPs was overlapping (Fig. 10C).

## Discussion

Tumor metastasis is the leading cause of death in cancer patients [57]. Many methods have been developed to prevent tumor metastasis; however, many patients fail to benefit from these methods, because they have already developed tumor metastasis at the time of diagnosis [58]. Some cancers preferentially metastasize to the lymph nodes. The accurate identification of SLN with tumor metastasis is crucial for diagnosis and treatment. A positive SLN is likely to indicate tumor dissemination to distant lymph nodes and organs. Patients with negative SLN are excluded from subsequent lymph node metastases [59]. Not all patients with possible lymph node metastasis require regional lymph node dissection. Several clinical trials in patients with melanoma have shown that regional lymph node dissection does not significantly improve patient outcomes compared with sentinel lymph node biopsy [60, 61]. ICG, the most widely used NIR dye approved by the FDA, has already been used to identify SLN and has progressed to clinical trials [62–64]. Although ICG has high sensitivity when used as a contrast agent to identify SLN, its specificity is not ideal [64]. In our study, nano-loaded ICG was applied to improve the optical stability of ICG and peptide modification was used to enhance its specificity [43, 65].

Compared with inorganic nanomaterials, such as nano-gold, nano-silicon, and nano-carbon, polymer materials have better biosafety, which indicates their greater application potential in drug delivery and clinical practice [38, 40, 66]. PLGA is recognized as one of the most biodegradable polymer materials currently synthesized, with good controllability, biodegradability, and biocompatibility [67, 68]. In our study, PEG-PLGA was used to carry ICG. PEG-PLGA, obtained by linking PLGA and PEG, is an amphiphilic polymer with good biodegradability and biocompatibility. It is a thermodynamically stable system that encapsulates hydrophobic drugs in a hydrophobic core for solubilization. Indeed, an appropriate particle size can ensure penetration and retention effects in tumor diagnosis and treatment. Meanwhile, PEGylation prevents phagocytosis by the reticuloendothelial system to prolong the blood circulation time [69]. In addition, it is relatively easy to modify proteins, antibodies, and polypeptides on the polymer surface, as indicated by the synthesis of PEG-PLGA polymers modified with hyaluronic acid (HA) and CD44 [70, 71]. We modified PEG-PLGA with MBA to enable linking with the polypeptide TMVP1 [31]. PEG-PLGA-packaged ICG showed improved stability and increased circulation time in the bloodstream, compared to free ICG thus creating an opportunity for EPR.

There is increasing evidence showing that tumor-induced lymphangiogenesis is a reliable predictor of lymph node metastasis and a promising target for metastasis prevention [72]. Tumors can express growth factors, including VEGF-A, VEGF-C, and VEGF-D, to induce lymphangiogenesis and promote the occurrence of metastasis in draining lymph nodes and other sites. VEGFR-3, as a specific receptor for VEGF-C and VEGF-D, participates and plays key roles in these processes. During the fetal period, VEGFR-3 is highly expressed in vascular endothelial cells before lymphatic differentiation, and is gradually restricted to lymphatic endothelial cells after the second trimester. Finally, VEGFR-3 expression is restricted to the new lymphatic endothelial system in adult tissues [73–75]. Research indicates that vascular remodeling in tumor-metastatic lymph nodes may occur ahead of tumor metastasis [4, 76]; therefore, VEGFR-3, as a marker of new lymphatic vessels, is appropriate as a molecular imaging ligand to predict and indicate metastasis [37, 77]. This study focused on the expression of VEGFR-3 in tumor-induced new lymphatic vessels. We used the peptide TMVP1, a ligand of VEGFR-3, screened using bacterial flagellin peptide library technology in our laboratory, to target tumor-induced new lymphatic vessels. The conjugate was attached to ICG-encapsulated PEG-PLGA micelles to image VEGFR-3 expression in SLN, suggesting the occurrence of tumor metastasis in the SLN. This is a type of active targeting molecular imaging. Currently, there are no known lymph node marker molecules. Previous studies on SLN imaging used the EPR of nanoparticles and the physicochemical properties in the tumor microenvironment showed poor specificity compared to active targeting [78, 79]. TMVP1-ICG-NPs are the nanoprobes to specifically target new lymph vessels during SLN metastasis.

With the involvement of photosensitizers, image-guided PTT and PDT are becoming increasingly attractive owing to their advantages, such as non-invasiveness, outstanding specificity, effective tumor ablation, and less side targeting [35, 39]. An NIR laser with strong tissue-penetrating capability is suitable for the treatment of solid tumors. PTT can damage the cell membrane, destroy the cytoskeleton, and inhibit DNA synthesis, thereby killing tumor cells. The release of ROS causes mitochondrial dysfunction and triggers apoptosis. PDT can generate singlet oxygen, which causes the apoptosis, autophagy, and necrosis of tumor cells. In this study, nanoparticles provided a passive target for the photosensitizer ICG, and TMVP1 provided an active target for ICG. TMVP1-ICG-NPs exhibited good photothermal and photodynamic effects. They significantly inhibited tumor growth under NIR laser irradiation, without any side effects. This is a practical option for imaging-guided therapy. Furthermore, tumor ablation after PTT and PDT can elicit an immune response by releasing tumor-associated antigens and activating systemic antitumor immune responses. Nanoparticles are also a suitable platform for the delivery of autologous antigens when to perform a combination therapy [80]. However, a single PTT/PDT treatment cannot cure a tumor, as the inhibited tumor may recur over time. Image-guided PTT and PDT may be the optimal choice for combination therapy [81].

In this study, we designed TMVP1-modified ICG-encapsulated PEG-PLGA nanomicelles that enable the use of TMVP1-ICG-NPs for molecular imaging by binding to VEGFR-3. TMVP1-ICG-NPs possess suitable physical and chemical properties and show good tumor site-specific aggregation properties in mouse breast cancer 4T1 cell and human cervical epidermoid carcinoma MS751 cell subcutaneous tumor-bearing mice [82]. Assessment of the use of TMVP1-ICG-NPs for PTT and PDT showed good inhibition in 4T1-tumor-bearing mice. Therefore, TMVP1-ICG-NPs may be used as an adjuvant therapy when performing combination therapy. The expression of VEGFR-3 in the SLN of tumor metastases showed a tubular pattern, which was consistent with the course of new lymphatic vessels, indicating that TMVP1-ICG-NPs targeting VEGFR-3 could efficiently image metastatic sentinal lymph nodes, also the specific expression of VEGFR-3 on new lymphatic vessels could provide higher specificity than other tumor metastasis targeting peptide [83, 84]. The polymer used in this study has been approved by the FDA for its safety and degradability, making it potentially suitable for clinical applications than other inorganic material [85]. We summarized recent research in the field of nanomedicine on targeted SLN imaging and PTT at Table 2. Also, the preparation process of TMVP1-ICG-NPs was simple and easy to store and volume production. Subsequently, testing the imaging capability of TMVP1-ICG-NPs in other tumors and clinical trials in patients are expected to be performed in the future.

## Conclusions

In summary, we successfully developed a novel NIR probe for SLN imaging and imaging-guided PTT and PDT. TMVP1-ICG-NPs is a novel tumor-targeting nanoprobe that can specifically bind to VEGFR-3 *in vivo *to indicate the occurrence of tumor metastasis in SLN and can be used for PTT and PDT to destroy the tumor. Our innovation TMVP1 was expectantly used as an active targeting molecular probe with highly specific. As research on the applications of nanomedicine in tumor diagnosis and treatment are becoming increasingly abundant, the accurate identification of tumor metastases in SLN at the molecular level will help realize the individualized treatment of patients with tumors. In addition, as a novel molecular imaging theranostic nanomedicine, TMVP1-ICG-NPs have high safety and stability, with good clinical translation prospects. Yet we lack the search for positive thresholds, further studies are required to compare and quantify the targeting ability of TMVP1-ICG-NPs in multiple models and stages to confirm the sensitivity and specificity.

**Table 2:** The nanomedicine research of SLN imaging and PTT

Nanoparticle	Material	Method	Function	Mechanism	Ref.
Ta@PVP NPs	Poly(vinylpyrrolidone)	Wet-ball milling method	Photoacoustic imaging PTT	EPR	[86]
SF@ICG NPs	Natural silk fibroin	Nanoprecipitation	NIR-II imaging PTT	EPR	[87]
N4-cRGD NPs	DSPE-PEG	Nanoprecipitation	Photoacoustic imaging PTT	EPR; RDG/ integrin αvβ_3_	[83]
[^64^Cu]-labeled PDI NPs	Perylene Diimide (PDI)	Nanoprecipitation	Photoacoustic imaging Positron Emission Tomography (PET)imaging PTT	EPR	[88]
AINP	Ancient ink	Modified hydrothermal reaction	Photoacoustic imaging PTT	EPR	[89]
CuS − DOX − Nd/FA NPs	Copper (II) chloride	Covalently conjugated; Self- assembly	NIR-II imaging PTT/chemotherapy	EPR; FA / FR	[90]
CNPs	Carbon nanoparticles	Modified three-step emulsion process	Photoacoustic and ultrasound imaging PTT	EPR	[91]
AuNRs@RGD	Gold nanoparticle	Seedless growth technique	Photoacoustic imaging Plasmonic Photothermal Therapy (PPTT)	EPR; RGD/ integrin αvβ_3_	[92]
TMP	Pluronic F127	Surfactant-stripping approach	PTT	EPR; Trastuzumab/HER2	[84]
RGD-CuS-Cy5.5	Copper sulfide PEG_2000_	Covalently conjugated	Multimodality imaging NIR/CT PTT	EPR; RGD/αvβ_3_	[85]

### Materials

NH_2_-PEG-NH_2_(MW, 3500) was purchased from Beijing Kaizheng Bioengineering Corporation (Beijing, China). PLGA-COOH (50/50; MW, 10,000) was obtained from the Shandong Medical Device Research Institute (Jinan, China). Poly (lactide-co-glycolide) (polyethylene glycol) Methoxy (MPEG (MW, 5,000)-PLGA (50/50; MW, 20,000)) was purchased from Jinan Daigang Biomaterial Co. Ltd. (Jinan, China). Dichloroethane (EDC), N-hydroxysuccinimide (NHS), 4-Maleimidobutyric Acid (MBA), and ICG were purchased from Sigma-Aldrich (St. Louis, MO, USA). N, N-dimethylformamide (DMF), dichloromethane (DCM), diethyl ether, and methanol were purchased from Aladdin Reagents (Shanghai, China). The cyclic polypeptide cTMVP1 (LARGRC, end-collateral amide bond into a ring, with one cysteine residues to provide the sulfhydryl group to react with MBA) was synthesized by Wuhan Tanda Scientific Co., Ltd. (Wuhan, China), and the compounds were > 95% pure by HPLC and MS analysis (Figure S1 and S2).

## Methods

### Synthesis of ICG-loaded PEG-PLGA micelles modified with TMVP1 and control nanoprobes

The synthesis method of TMVP1-PEG-PLGA has been described in our previous study [31, 93]. The synthesis flow is shown in Fig. 1. First, MBA was activated at room temperature for 5 h, together with EDC and NHS. PEG and triethylamine were then added to the reaction system for 48 h at room temperature to obtain maleimide-bond-terminated polyethylene glycol (Mal-PEG)[94]. Next, PLGA, EDC, and NHS were dissolved in DCM, activated at room temperature for 5 h, and then added to the reaction system and reacted at room temperature for 48 h to obtain Mal-PEG-PLGA. Finally, the above products were dropwise added into the mixture solution (ratio of diethyl ether/methanol was 5/1), the precipitate was washed with the mixture solution twice, and the final Mal-PEG-PLGA product was dried in a vacuum for 12 h. The TMVP1 peptide and Mal-PEG-PLGA (1:20, w/w) were dissolved in 800 µL of DMF solution, with continuous stirring, at room temperature for 8 h to obtain TMVP1-PEG-PLGA. ICG-loaded PEG-PLGA micelles were prepared using a self-assembly method [87]. Two hundred micrograms of free ICG was added to the 800 µL DMF solution of TMVP1-PEG-PLGA (20 mg/mL). This mixture was added dropwise under stirring to 15 mL of deionized H_2_O. After 30 min, the solution was centrifuged for 30 min at 13,000 rpm to collect the nanoprobes, which were washed twice with deionized H_2_O. The final nanoprobe was redispersed by sonication in 1 mL of water. ICG-NPs micelles were prepared similarly, except that TMVP1-PEG-PLGA was replaced with MPEG-PLGA.

### Physical and analytical method

Room temperature FT-IR spectra of synthetic samples were determined on a Perkin Elmer Spectrum RX I spectrophotometer using KBr pellets. ^1^HNMR spectra were carried out on a Bruker Avance NEO-600 spectrometer at room temperature using DMSO-d6.

### Characteristics of the NPs

The morphology of the NPs was determined using TEM (JEM-1230; JEOL, Tokyo, Japan). The particle sizes and zeta potentials of the NPs were determined using DLS (Zeta Plus, Brookhaven Instruments, USA). A UV-Vis-NIR spectrophotometer (PerkinElmer, Waltham, MA, USA) was used to measure the absorbance of ICG at 780 nm. The ICG in the supernatant from the nanofabrication process was measured and used to calculate the encapsulation efficiency. The encapsulation efficiencies (EE) of ICG were calculated according to the following formula: EE (%) = (ICG_Total_ - ICG_Super_)/ICG_Total_ × 100%.

### Cells and animals

The mouse breast cancer cell line, 4T1; the human cervical cancer cell line, MS751; and the human keratinocyte cell line, HaCaT, were purchased from the American Type Culture Collection (Manassas, VA, USA). 4T1 cells were cultured in RIPA-1640 medium supplemented with 10% fetal bovine serum (FBS). MS751 and HaCaT cells were cultured in Dulbecco’s modified Eagle’s medium supplemented with 10% FBS. Both media were supplemented with 100 IU/mL penicillin and 100 µg/mL streptomycin (Invitrogen, Carlsbad, CA, USA). Female 4-week-old BALB/c mice and BALB/c nude mice were purchased from Jicui Yaokang Biological Technology Co., Ltd. (Nanjing, China). All animals were acclimated for 1 week before beginning the experiments under specific-pathogen-free conditions at the Experimental Animal Center of Tongji Hospital of Huazhong University of Science and Technology (HUST). All animal procedures were approved by the HUST Ethics Committee for Animal Experiments.

### ***In vitro*** cytotoxicity

CCK8 assays were performed to estimate the cytotoxicity of TMVP1-ICG-NPs. Five thousand for 4T1, MS751, and HaCaT cells were seeded into 96-well plates, and the media were replaced with 100 µL of serum-free culture medium containing different ICG equivalent concentrations [54] (200 µg/mL, 100 µg/mL, 50 µg/mL, 25 µg/mL, 12.5 µg/mL, 6.25 µg/mL, 3.125 µg/mL, or 0 µg/mL) of TMVP1-ICG-NPs. After incubation for 24 h, cells were washed three times with PBS and 90 µL of fresh medium and 10 µL of CCK8 solution (Dojindo Molecular Technologies, Kumamoto, Japan) were added. The cell plates were incubated for another 2 h, and the absorbance was then measured at 450 nm using a microplate reader (Bio-Rad, Hercules, CA, USA). Cell viability was calculated according to the following formula: cell viability (%) = (OD_Treat_ - OD_Blank_) / (OD_Control_ - OD_Blank_) × 100%, where OD is the optical density at 450 nm.

### Cellular uptake ***in vitro***

4T1 and MS751 cells were transfected with a lentivirus vector containing VEFGR-3 or a control vector. VEGFR-3 expression was detected using western blotting. VEGFR-3-overexpressing cells (4T1-Flt4 and MS751-Flt4 cells) were cultured in 12-well plates at a density of 1.0 × 10^5^ cells/well. TMVP1-ICG-NPs, ICG-NPs, or free ICG (100 ng/mL) were then added and the cells were incubated for 0.5 h, 1 h, 2 h, and 3 h. Flow cytometry was used to detect the fluorescence intensity, as an indicator of cellular uptake. The cells were seeded onto sterilized glass slides in 12-well plates at a density of 5 × 10^4^ cells/well. The NPs and free ICG were added and the cells were incubated for 2 h. The cells were then fixed with 4% paraformaldehyde for 10 min, and the cell nuclei were stained with 4′,6-diamidino-2-phenylindole. Images of cellular uptake were obtained using a fluorescence microscope (BX51; Olympus, Tokyo, Japan).

***In vivo*** **bio-distribution of TMVP1-ICG-NPs and pharmacokinetic study**

Normal BALB/c mice were used to investigate the dynamics and bio-distribution of the TMVP1-ICG-NPs. A TMVP1-ICG-NPs dose of 1 mg/kg was intravenously injected into mice and monitored using an IVIS imaging system (PerkinElmer) at excitation and emission wavelengths of 745 and 840 nm, respectively, for 48 h at different time points (0.5 h, 1 h, 2 h, 12 h, 24 h, 48 h). All fluorescence images were acquired by automatically subtracting the background and were displayed using the same color scale. The mice were euthanized by cervical dislocation, and the excised organs (heart, liver, spleen, lungs, kidneys, and intestines) were imaged at the corresponding time points. The region of interest (ROI) tool of Living Image® software 4.0 (PerkinElmer) was applied to determine the exact fluorescence radiant efficiency of the organs. Each group consisted of three mice.

SD rats were used for pharmacokinetic studies. Six SD rats were randomly divided into the following two groups: free ICG and TMVP1-ICG-NPs. The rats were intravenously administrated free ICG or TMVP1-ICG-NPs at a dose of 500 µg of equivalent ICG. At preset time points (10 min, 30 min, 1 h, 2 h, 4 h, 8 h, 12 h, 24 h, 48 h, and 72 h), 300 µL blood samples were collected into tubes containing EDTA and centrifuged at 1,500 rpm for 10 min to separate the plasma. The NIR fluorescence intensity of the plasma samples was measured using an IVIS imaging system (excitation/emission, 745/840 nm). Plasma concentrations of TMVP1-ICG-NPs and free ICG were calculated by comparison with a standard curve. The standard curve was obtained by preparing a series gradient of ICG concentrations (2 µg/mL, 1 µg/mL, 0.5 µg/mL, 0.25 µg/mL, 0.125 µg/mL, 0.05625 µg/mL, 0.028125 µg/mL, 0.0140625 µg/mL, 0.00703125 µg/mL, 0.003515625 µg/mL, 0.001757813 µg/mL, and 0.000878906 µg/mL) dissolved in rat plasma.

### ***In vivo*** tumor and SLN metastasis targeting ability

4T1 and MS751 tumor-bearing mice were used to evaluate the targeting ability of TMVP1-ICG-NPs *in vivo*. The mouse breast cancer cell line, 4T1, and the human cervical cancer lymph node metastasis cell line, MS751, were transfected with a luciferase lentivirus. 4T1-luc cells (1 × 10^6^ cells) were inoculated into the left hips of BALB/c mice, and 1 × 10^6^ MS751-luc cells were inoculated into the left hips of BALB/c null mice. The mice were imaged under an IVIS imaging system using a luminescent imaging model involving the intraperitoneal injection of 200 µL of luciferin substrate after approximately 2 weeks to confirm the tumor volume. When the tumor volume was approximately 150–200 mm^3^, the mice were intravenously administered ICG-NPs or TMVP1-ICG-NPs at a dose of 1.0 mg/kg equivalent ICG (six mice per group). The mice were then anesthetized and imaged using the IVIS imaging system at different time points (30 min, 1 h, 2 h, 4 h, 12 h, 24 h, and 72 h) using excitation/emission wavelengths of 745/840 nm. Three mice from each group were sacrificed 24 h post-injection to explore the tissue distribution of TMVP1-ICG-NPs in tumor-bearing mice. Tumors and major organs (heart, liver, spleen, lungs, kidneys, and intestines) were harvested for *ex vivo* imaging. The NIR fluorescence intensity was measured using the ROI tool of Living Image® software 4.0.

For the SLN metastasis model, 5 × 10^4^ 4T1-luc cells and 1 × 10^5^ MS751-luc cells were resuspended in 25 µL of PBS and injected into the foot pads on both sides of BALB/c mice and BALB/c null mice separately. Popliteal lymph node metastasis appeared at approximately 4 weeks. Mice with bilateral popliteal lymph node metastases and normal mice were injected with ICG-NPs and TMVP1-ICG-NPs via the foot pad, at a dose of 0.1 mg/kg equivalent ICG. ICG-NPs were injected into the left foot pad and TMVP1-ICG-NPs were injected into the right foot pad (six mice per group). NIR fluorescence was measured *in vivo* at designated time points (10 min, 30 min, 2 h, 4 h, 8 h, 12 h, and 24 h). Three mice from each group were sacrificed 4 h post-injection to obtain bilateral popliteal lymph nodes for NIR imaging. Lymph nodes were frozen in liquid nitrogen after image acquisition for immunofluorescence analysis of VEGFR-3. The NIR fluorescence intensity was measured using the ROI tool of Living Image® software 4.0.

### Verification of PTT and PDT using TMVP1-ICG-NPs

The photothermal and photodynamic effects of ICG were evaluated in 24-well plates. One milliliter of 10 µg/mL TMVP-ICG-NPs was added to a 24-well plate and irradiated using a NIR laser (HU80513, 808 nm; Changchun, China) at power levels of 0.5 W/cm^2^, 1 W/cm^2^, 1.5 W/cm^2^, 2 W/cm^2^, and 2.5 W/cm^2^ for 10 min. Temperature changes were measured using an infrared thermometer. A power of 2.5 W/cm^2^ was used to determine the photothermal effects of different ICG concentrations. The fluorescence intensity of a single oxygen molecule was detected using 2,7-dichlorofluorescin (MX4803; MKBio, Shanghai, China). PBS, 10 µg/mL ICG, and ICG-NPs and TMVP1-ICG-NPs with 10 µg/mL equivalent ICG were added with 1 µM 2,7-dichlorofluorescin and irradiated with an NIR laser (808 nm, 2.5 W/cm^2^). Fluorescence was then detected at excitation/emission wavelengths of 504/529 nm (Infinite M1000; Tecan, Männedorf, Switzerland). Using the same method, the above mentioned groups were irradiated and the temperature increase was detected using an infrared thermometer. 4T1-Flt4 cells (3 × 10^4^ cells) were seeded into 24-well plates to examine their ROS content. Free ICG or ICG-NPs or TMVP1-ICG-NPs with 10 µg/mL equivalent ICG were added to the 24-well plates for 3 h, after which, 200 µL of a dihydroethidium solution (5 µM in as) was added and the cells were irradiated at 808 nm (2.5 W/cm^2^) for 10 min. Fluorescent images were obtained at excitation/emission wavelengths of 518/610 nm using an Olympus IX71 microscope. 4T1-Flt4 cells (5 × 10^3^ cells) were seeded in 96-well plates to evaluate cell viability after NIR laser irradiation with the following groups: free ICG + laser, ICG-NPs, ICG-NPs + laser, TMVP1-ICG-NPs, TMVP1-ICG-NPs + laser at concentrations of 2 µg/mL, 5 µg/mL, 8 µg/mL, and 10 µg/mL. 4T1-Flt4 cells (1 × 10^5^) cells were seeded into 12-well plates to examine apoptosis. Free ICG or ICG-NPs or TMVP1-ICG-NPs with 10 µg/mL equivalent ICG were added to the cells for 3 h. The cells were then irradiated at 808 nm (2.5 W/cm^2^) for 10 min and detected by flow cytometry.

Mice bearing subcutaneous 4T1-luc tumors were randomly divided into six groups (PBS, free ICG + laser, ICG-NPs, ICG-NPs + laser, TMVP1-ICG-NPs, and TMVP1-ICG-NPs + laser) when the tumor volume reached approximately 100 mm^3^. The mice were intravenously administered PBS, ICG, ICG-NPs, or TMVP1-ICG-NPs at a dose of 1 mg/kg. The mice in the laser treatment group were irradiated with an NIR laser for 5 min (808 nm, 2.5 W/cm^2^) 24 h post-injection. The mouse tumor volume (V = L × W^2^/2) and body weight were measured every 2 d. The tumors were monitored using luminescence imaging every week.

### ***In vivo*** safety analysis

Toxicity studies were performed using 6-week-old BALB/c mice and 10 times the ICG imaging dose (10 mg/kg) of TMVP1-ICG-NPs. The same volume of PBS was injected into the tail vein as a control. The mice were observed every 2 d, and their body weight was measured. At 28 d post-injection, all of the mice were sacrificed. Blood samples were collected for cell counting and the detection of biochemical indicators, including ALT, AST, BUN, UR,and CR. The main organs (heart, liver, spleen, lungs, and kidneys) were stained with hematoxylin and eosin for histopathological analysis.

### Immunohistochemistry

Mouse specimens were embedded in paraffin and sectioned to a thickness of 4 mm for immunohistochemical staining. In brief, after the tissue sections were subjected to antigen retrieval and blocking with 5% bovine serum albumin, they were incubated with primary antibody at 4℃ overnight. A primary antibody against VEGFR-3 (552,857, 1:50; BD Biosciences, US) was used. The following day, the tissues were incubated with secondary antibody for 1 h at 37℃. Tissue staining was performed using a 3,3′-diaminobenzidine kit (G1212; Servicebio, Wuhan, China). Immunofluorescence was performed as described above, using a fluorescent secondary antibody.

### Statistical analysis

Statistical analyses were conducted using Prism software (version 6.0; GraphPad, San Diego, CA, USA). Data were analyzed using an unpaired Student’s t-test (two groups) or analysis of variance (three or more groups).

## Electronic supplementary material

Below is the link to the electronic supplementary material.

Supplementary Material 1

## Notes

The original online version of this article was revised: Underline in the text has been removed.
