# Supplementary material for: A TMVP1-modified near-infrared nanoprobe: molecular imaging for tumor metastasis in sentinel lymph node and targeted enhanced photothermal therapy
Source: J Nanobiotechnology. 2023 Apr 17;21:130. doi: 10.1186/s12951-023-01883-6 (PMC10108508; doi:10.1186/s12951-023-01883-6)
Supplement: Supplementary file 1 — Supplementary Material 1 [file 12951_2023_1883_MOESM1_ESM.docx]

**Supporting Information**

**TMVP1-modified near-infrared nanoprobe for tumor metastasis SLN imaging and enhanced PTT by binding to VEGFR-3**

Xueqian Wang ‡ ^1,2^, Geyang Dai ‡ ^1,2^, Guiying Jiang^3^, Danya Zhang^1,2^, Ling Wang^1,2^ Wen Zhang^5^, Huang Chen^6^, Teng Cheng^7^, Ying Zhou^1,2^, Xiao Wei^1,2^, Fei Li^1,2^, Ding Ma^1,2^, Songwei Tan^4^, Rui Wei *^1,2^, Ling Xi *^1,2^

^1^ Department of Gynecological Oncology, Tongji Hospital, Tongji Medical College, Huazhong University of Science and Technology, Wuhan 430000, China

^2^ National Clinical Research Center for Obstetrics and Gynecology, Cancer Biology Research Center (Key Laboratory of the Ministry of Education), Tongji Hospital, Tongji Medical College, Huazhong University of Science and Technology, Wuhan 430000, China

^3^ Department of Gynecology, West China Second University Hospital, Chengdu 610000, China

^4^ Tongji School of Pharmacy, Tongji Medical College, Huazhong University of Science and Technology, Wuhan 430000, China

^5^ Hubei University of Medicine, Shiyan 442000, China

^6^ School of Medicine, Jianghan University, Wuhan 430000, China

^7^ Department of Thyroid and Breast Surgery, Tongji Hospital, Tongji Medical College, Huazhong University of Science and Technology, Wuhan, 430030, China.

‡ These two authors contributed equally to this work

*Correspondence: Ling Xi, [lxi@tjh.tjmu.edu.cn](mailto:lxi@tjh.tjmu.edu.cn); Rui Wei, [weirui2018@tjh.tjmu.edu.cn](mailto:weirui2018@tjh.tjmu.edu.cn)


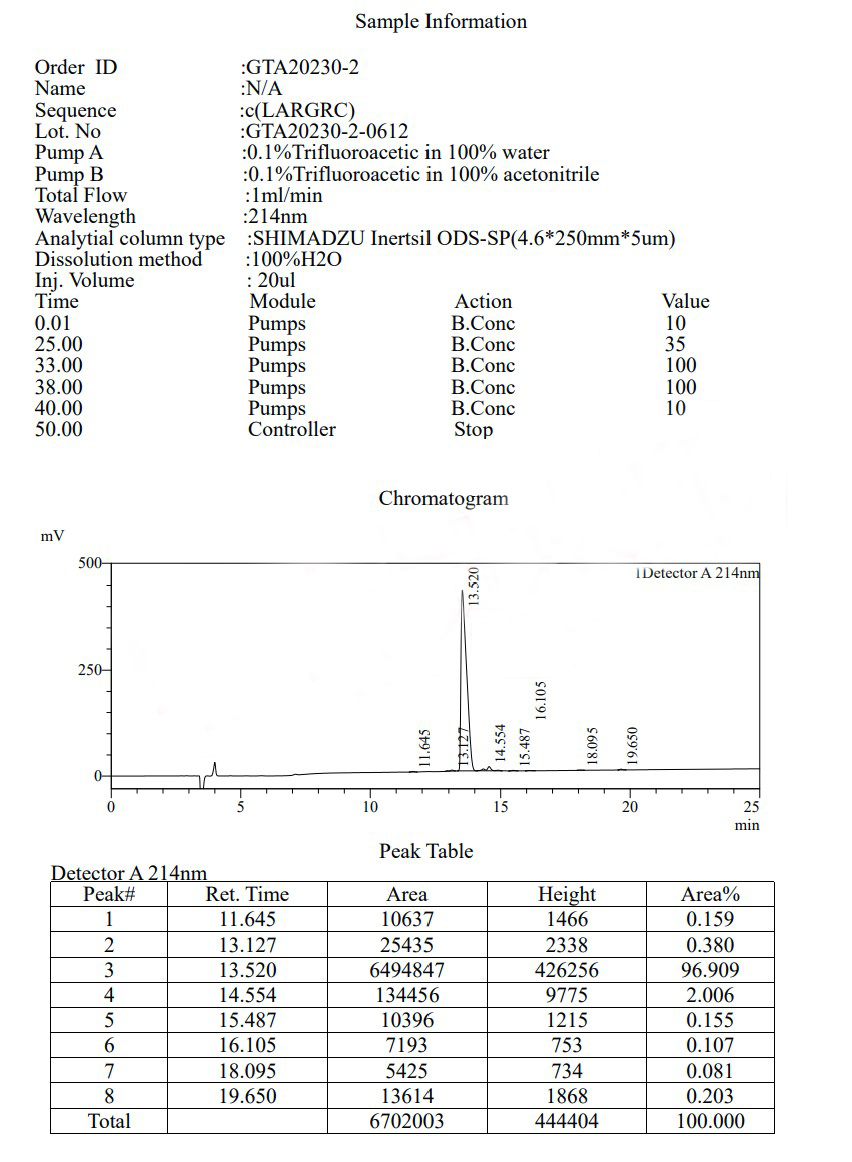


**Figure S1** HPLC of cTMVP1, the purity of cTMVP1 was 96.909%.


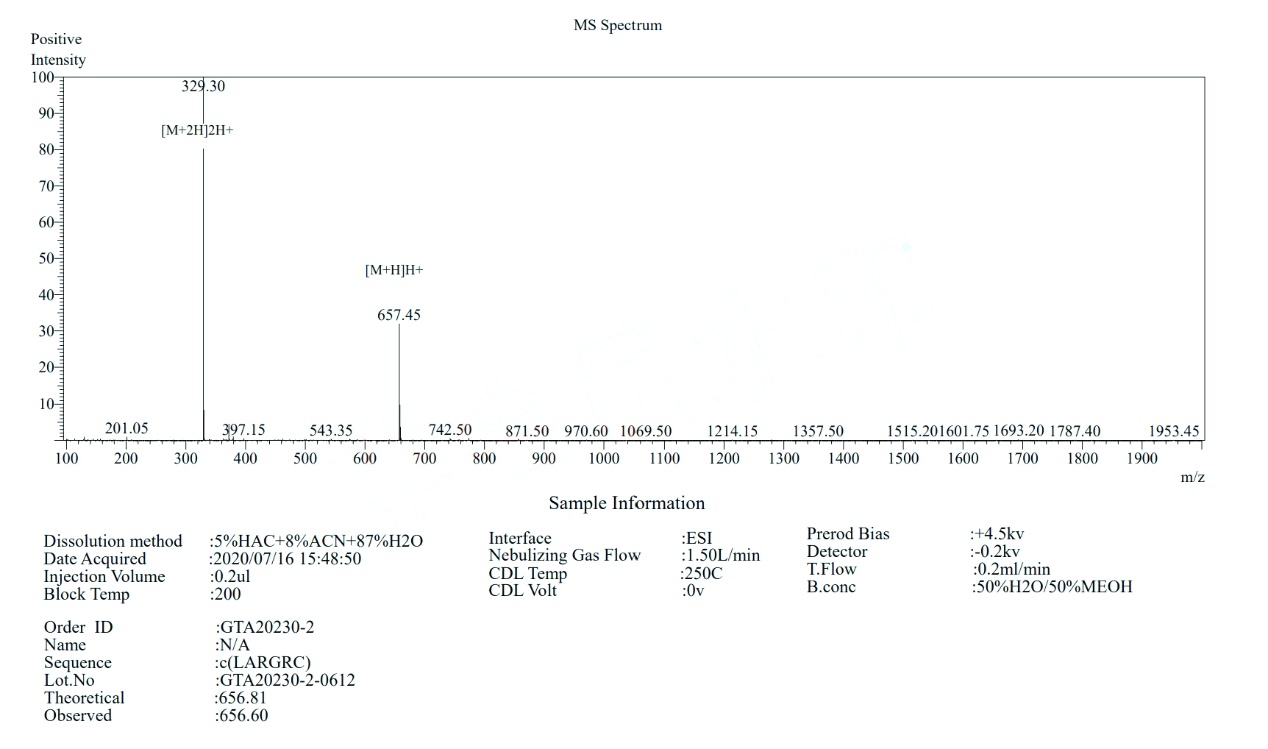


**Figure S2** MS spectrum of cTMVP1.


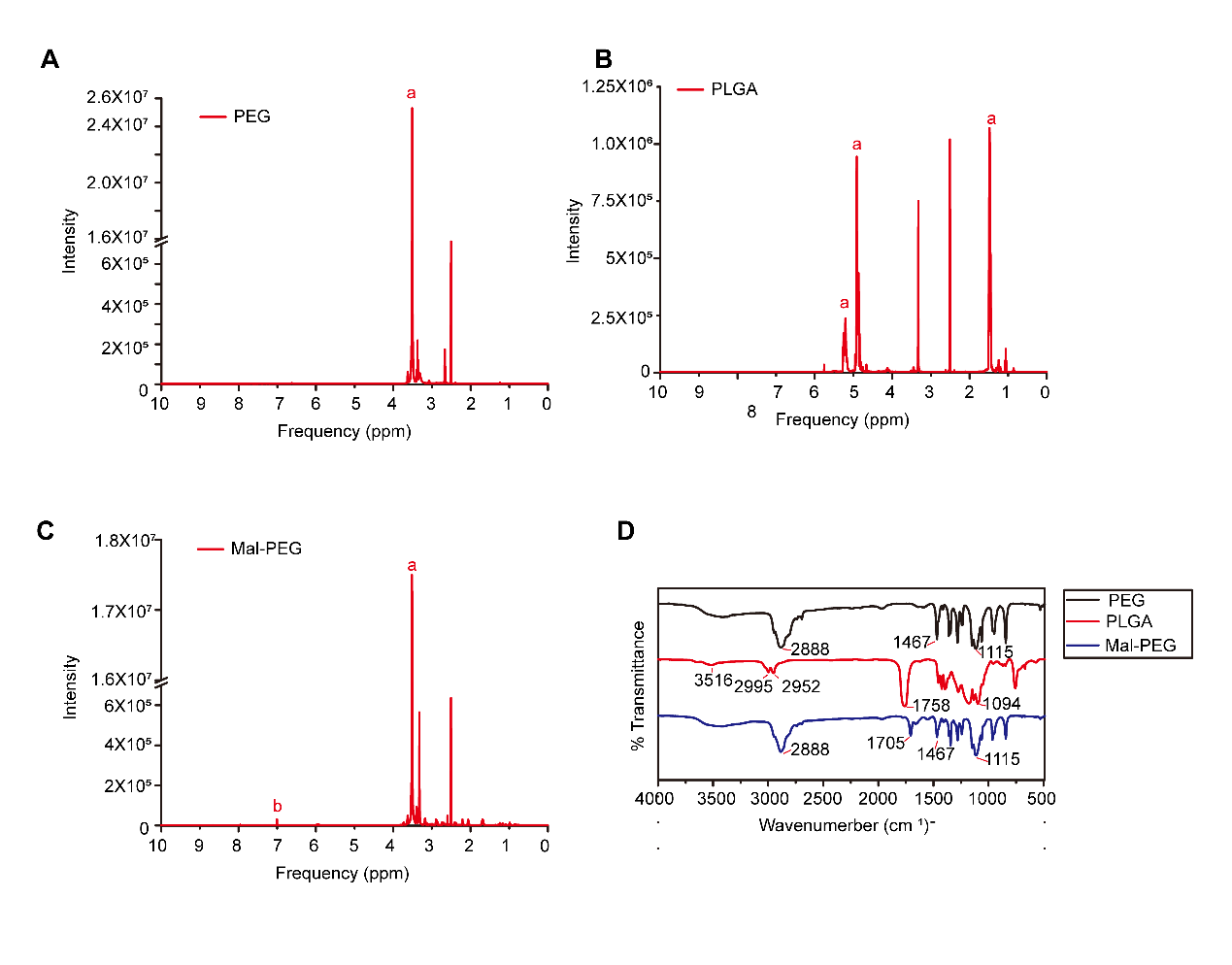


**Figure S3** ^1^HNMR and FT-IR of PEG, PLGA and Mal-PEG. (A) ^1^HNMR spectra of PEG, the absorption peak at 3.51 ppm (a) represent the -CH_2_ peak of PEG. (B) ^1^HNMR spectra of PLGA, the absorption peak at 5.21 ppm, 4.92 ppm, and 1.48ppm (a) represent the -CH_3_, -CH_2_, and -CH of PLGA. (C) ^1^HNMR spectra of Mal-PEG, the absorption peak at 3.51 ppm (a) represent the -CH_2_ peak of PEG, and the peak at 7.00 ppm (b) was represent maleimide bond. (D) FT-IR spectra of PEG, PLGA and Mal-PEG. The characteristic absorbed bonds of PEG appeared at 2888cm^-1^ (-CH_2_), 1467cm^-1^ (-NH_2_), 1115cm^-1^ (-C-O). The characteristic absorbed bonds of PLGA appeared at 3516cm^-1^ (-OH), 2995-2952cm^-1^ (-CH_2_, -CH_3_), 1467cm^-1^ (-NH_2_), 1115cm^-1^ (-C-O). The characteristic absorbed bonds of Mal-PEG appeared at 2888cm^-1^ (-CH_2_), 1467cm^-1^ (-NH_2_), 1115cm^-1^ (-C-O), and 1705 cm^-1^ (-C=C).


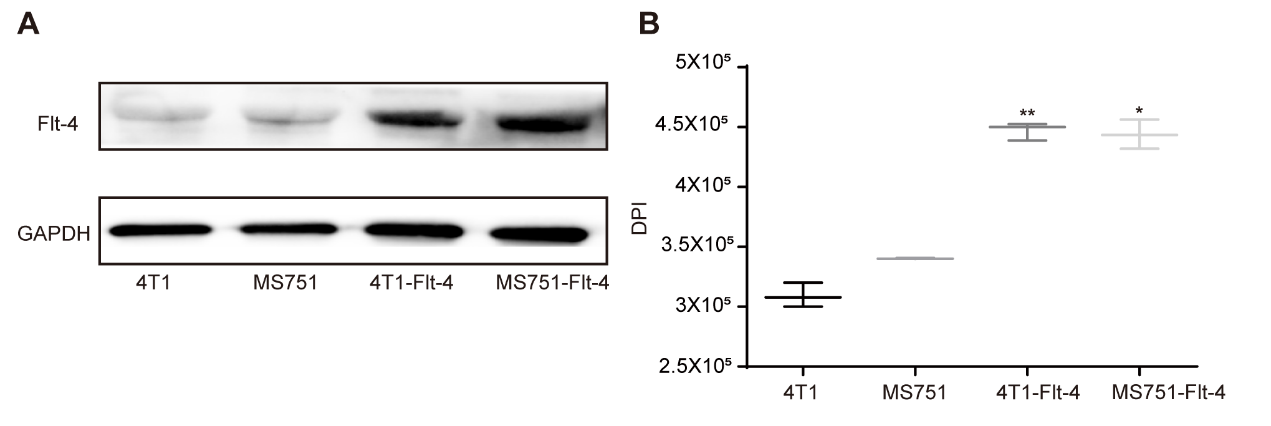


**Figure S4** Expression of VEGFR-3. (A) Western blot of VEGFR-3. (B) Gray value statistics of A.
